# Targeted nanovesicular delivery of dexmedetomidine modulates microglial lysosomal function via Sirt3 signaling to ameliorate neurodegenerative pathology

**DOI:** 10.4103/NRR.NRR-D-25-00234

**Published:** 2026-02-28

**Authors:** Yuan Zhang, Hui Jiang, Fuqing Zhang, Jinghua Liao

**Affiliations:** Department of Anesthesiology, Clinical Oncology School of Fujian Medical University, Fujian Cancer Hospital, Fuzhou, Fujian Province, China

**Keywords:** Alzheimer’s disease, amyloid-beta, aptamer, cognitive impairment, dexmedetomidine, drug delivery, extracellular nanovesicles, lysosome, microglia, neuroinflammation, Sirtuin 3

## Abstract

Neuroinflammation and lysosomal dysfunction in microglia are increasingly recognized as critical pathological drivers of Alzheimer’s disease, yet current anti-inflammatory or neuroprotective agents have limited brain delivery efficiency and cellular specificity. To address these challenges, this study proposes a novel nanotherapeutic paradigm based on extracellular nanovesicles (ENVs) for targeted modulation of microglial function. This research explored the potential of a novel nanotherapeutic platform involving ENVs functionalized with aptamers and encapsulating dexmedetomidine (Dex) to alleviate microglia-associated neuroinflammation in Alzheimer’s disease. The effects on microglial lysosomal function, neuroinflammation, and cognitive performance were evaluated in an Alzheimer’s disease mouse model. Cholesterol-conjugated PEG 2000 aptamers were used to modify extracellular nanovesicles derived from microglial cells. The nanovesicles (ZH-1c-ENVs) were loaded with Dex using ultrasound-assisted methods. Particle size, morphology, and drug release kinetics were characterized using dynamic light scattering and transmission electron microscopy. *In vitro* assays assessed microglial cell uptake and cytotoxicity, while *in vivo* biodistribution was evaluated in a mouse model. Proteomics, western blotting, quantitative reverse transcription-polymerase chain reaction, and immunofluorescence were employed to analyze the effects of ZH-1c-ENVs@Dex on microglial inflammation, lysosomal activity, and amyloid-beta clearance. Cognitive function improvements were assessed using the Morris water maze. ZH-1c-ENVs@Dex achieved efficient drug encapsulation and crossed the blood–brain barrier, delivering Dex selectively to microglial cells. Proteomic analysis revealed activation of the Sirtuin 3 signaling pathway, which reduced microglial inflammation and enhanced lysosomal function. These changes promoted amyloid-beta clearance *in vitro* and alleviated neuroinflammation *in vivo*. Treatment significantly improved cognitive performance in Alzheimer’s disease mice. The ZH-1c-ENVs@Dex system represents a promising nanomedicine strategy for Alzheimer’s disease therapy by modulating Sirtuin 3 activity, restoring microglial function, and improving cognitive outcomes. This study lays the groundwork for clinical translation of aptamer-modified ENVs as precision nanomedicines for neurodegenerative diseases. The ZH-1c-ENVs@Dex system integrates clinically safe components, efficiently traverses the blood–brain barrier, and selectively targets microglia, exhibiting remarkable potential for the treatment of Alzheimer’s disease and related neurodegenerative disorders. This scalable and highly biocompatible nanovesicular platform offers a clinically translatable strategy with substantial therapeutic promise for neuroinflammatory and neurodegenerative diseases.

## Graphical abstract

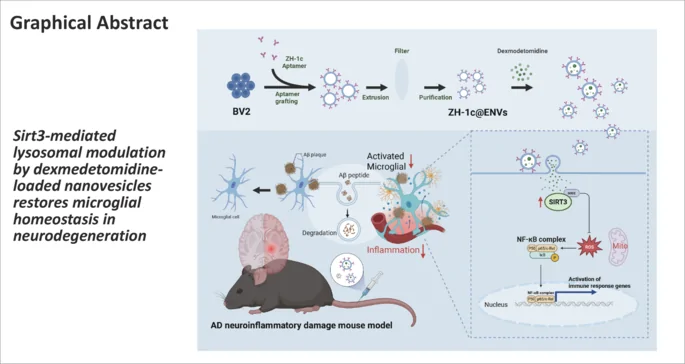

## Introduction

Alzheimer’s disease (AD) ranks among the most prevalent neurodegenerative disorders, severely affecting the quality of life in the global aging population. In recent years, the incidence of AD has continued to rise due to population aging, placing a growing burden on families and healthcare systems (Zhang et al., 2024a). The pathogenesis of AD is complex, involving multiple interacting factors, among which neuroinflammation is widely recognized as a critical component in its pathological progression (Thawabteh et al., 2024; Yang et al., 2024). Microglial cells, the primary immune effectors in the central nervous system (CNS), undergo excessive activation in response to pathological insults and release large quantities of inflammatory mediators. This exacerbates neuroinflammation, leading to neuronal death and progressive cognitive decline (Melchiorri et al., 2023; Chu et al., 2024). Current therapeutic strategies for AD mainly aim to alleviate symptoms and delay progression, but effective approaches for controlling neuroinflammation remain lacking (Dhapola et al., 2021). The development of targeted interventions that modulate inflammatory responses has emerged as an urgent therapeutic need (Fornari Laurindo et al., 2024).

In AD, microglial dysfunction is considered a key factor in neuroinflammation and impaired clearance of amyloid-beta (Aβ) deposits. Under normal conditions, microglia maintain the stability of the neural microenvironment by removing harmful substances in the central nervous system through lysosomal pathways (Sarlus and Heneka, 2017; Rangaraju et al., 2018). However, in AD patients, microglia frequently adopt a proinflammatory phenotype and exhibit pronounced lysosomal impairment (Gao et al., 2023; Sudwarts and Thinakaran, 2023; Lepiarz-Raba et al., 2024). This dysfunction not only reduces the efficiency of Aβ clearance but also exacerbates the inflammatory microenvironment, creating a vicious cycle (Cai et al., 2022; Miao et al., 2023; Twarowski and Herbet, 2023). Restoring lysosomal function and attenuating microglial inflammation have emerged as promising therapeutic strategies for AD (Lepiarz-Raba et al., 2024; Serradas et al., 2024). However, achieving precise regulation of microglial function remains a significant challenge and a focal point of current research (Althafar, 2022; McKee et al., 2023).

Extracellular nanovesicles (ENVs) have emerged as promising candidates for drug delivery in recent years, owing to their distinctive biological properties. ENVs are membrane-bound entities derived from cellular lipid bilayers, ranging in size from 30 to 300 nm (Alderton, 2015). Their intrinsic biocompatibility and low immunogenicity enable efficient penetration of the blood-brain barrier (BBB) and facilitate targeted drug delivery (Yin et al., 2023; Tsakiri et al., 2024; Yadav et al., 2024). Modification of ENVs with aptamers further enhances their targeting specificity and delivery efficiency, offering new therapeutic strategies for CNS disorders (Loch-Neckel et al., 2022; You et al., 2024). The structural versatility of aptamers allows for the integration of diverse functional molecules, making these systems especially suitable for selective regulation of specific cell types such as microglia (Ou et al., 2023; Chu et al., 2024). Consequently, aptamer-modified ENVs provide a robust foundation for drug delivery and target regulation in AD treatment (Bahadorani et al., 2024; Banik et al., 2024). Furthermore, ENVs have been developed as drug carriers that can fuse with cell membranes to directly deliver drugs into the cytoplasm, significantly improving the delivery efficiency of sensitive molecules by avoiding lysosomal degradation (Schiffelers et al., 2012; Ohno et al., 2013).

Dexmedetomidine (Dex), an α2-adrenergic receptor agonist, has emerged as a promising compound in neuroscience research due to its sedative, anti-inflammatory, and neuroprotective properties (Zhu et al., 2024). Studies have demonstrated that Dex significantly suppresses inflammatory responses and protects neurons (Wang et al., 2022). However, challenges remain in achieving targeted delivery and efficient utilization of Dex in the CNS (Burlacu et al., 2022; Krzyzaniak et al., 2023). Sirtuin 3 (Sirt3) is a mitochondrial deacetylase that serves as a key regulator of cellular redox balance and metabolic homeostasis and is also critically involved in modulating inflammatory pathways and lysosomal activity (Yang et al., 2020; Zhang et al., 2024b). Activation of the Sirt3 signaling pathway by Dex offers a potential mechanism for simultaneously suppressing microglial inflammation and restoring lysosomal function, thereby providing a promising therapeutic strategy for AD (Zhao et al., 2022; Qiao et al., 2023).

Building upon the above research background, this study aimed to synthesize and characterize ZH-1c aptamer-modified extracellular nanovesicles loaded with dexmedetomidine (ZH-1c-ENVs@Dex) and to investigate their therapeutic effects and underlying mechanisms in AD. Lipid conjugation technology was used to stably anchor the aptamer onto the surface of ENVs, thereby enhancing targeting specificity and uptake efficiency in microglial cells. Proteomic analysis was conducted to elucidate the molecular mechanisms by which ZH-1c-ENVs@Dex modulates the Sirt3 signaling pathway. Both *in vitro* and *in vivo* experiments were performed to investigate how ZH-1c-ENVs@Dex influences microglial inflammatory responses, lysosomal activity, and the clearance of Aβ deposits. Furthermore, its therapeutic efficacy in mitigating neuroinflammatory injury and improving cognitive performance was validated in an AD mouse model. This study not only introduces an innovative approach for the precise treatment of AD but also contributes to the advancement of extracellular nanovesicle-based drug delivery systems with broad scientific and translational potential.

## Methods

### Ethics statement

All animal experiments were conducted in strict accordance with international and national ethical guidelines for the care and use of laboratory animals. The experimental protocol was approved by the Animal Ethics Committee of Clinical Oncology School of Fujian Medical University, Fujian Cancer Hospital (approval No. IACUCFJMU 2024-Y-2143, approval date: October 8, 2024). Animals were housed and handled under conditions designed to minimize pain and distress, and humane endpoints were used for euthanasia at the conclusion of the study. Throughout the experimental process, ethical principles were rigorously observed to ensure humane treatment and compliance with institutional standards.

### Lipid selection

Fluorescein isothiocyanate (FITC)-labeled stearoyl-polyethylene glycol_2000_ (C18-PEG_2000_), distearoylphosphatidylethanolamine-polyethylene glycol_2000_ (DSPE-PEG_2000_), and cholesterol-polyethylene glycol_2000_ (chol-PEG_2000_) were dissolved in ethanol at a concentration of 1 mM. Each lipid probe (3 nmol) was added to 1 × 10^7^ BV2 cells (ATCC, Manassas, VA, USA, RRID: CVCL_0182) in 250 µL of PBS. The BV2 cell samples labeled with FITC-lipid probes were incubated at 4°C for 5 minutes and subsequently washed three times. Subsequently, cells were fixed, counterstained with 4′,6-diamidino-2-phenylindole (DAPI), and rinsed thoroughly. Finally, the cells were resuspended in 500 µL of PBS, and 20 µL of the suspension was placed onto slides for imaging.

### Synthesis of ZH-1c-PEG_2000_-Chol and labeling of BV2 cells

To synthesize ZH-1c-PEG_2000_-Chol, 100 nmol of disulfide-labeled ZH-1c aptamer was reduced with TCEP solution on ice for 30 minutes. The reduced aptamer was then incubated overnight at 4°C in HEPES buffer (Sigma-Aldrich, St. Louis, MO, USA) with 500 nmol of chol-PEG_2000_-maleimide (NanoCS, New York, NY, USA). Excess chol-PEG_2000_-maleimide was removed by filtration using a 5000 molecular weight cut-off (MWCO) filter at 10,000 × *g* for 15 minutes. The purified FITC-labeled ZH-1c-PEG_2000_-Chol was analyzed using MALDI-TOF-MS. For cell labeling, 1–5 nmol of FITC-ZH-1c-PEG_2000_-Chol was added to 250 µL of PBS containing 1 × 10^7^ BV2 cells. The mixture was incubated at 4°C for 5 minutes, followed by three PBS washes. Fluorescence intensity was measured utilizing an Infinite M200 microplate reader (Tecan, Männedorf, Switzerland).

### Preparation of ZH-1c-ENVs

ZH-1c-ENVs were generated as described previously (Lozano et al., 2022) with modifications. Briefly, BV2 cells were collected by centrifugation at 500 × *g* for 5 minutes after PBS washing and resuspended in PBS. The cell suspension was sequentially extruded through 10, 5, and 1 μm polycarbonate membranes (Whatman, 19 mm; 610010, Advanti Polar Lipids, Alabaster, AL, USA), with each membrane being passed 13 times. The resulting vesicles were purified using a 10% OptiPrep^TM^ (Stemcell Technologies) cushion layered over a 10%/50% iodixanol step gradient, followed by ultracentrifugation at 100,000 × *g* for 2 hours at 4°C. Seven equal fractions were collected, diluted to 1.5 mL with PBS, and centrifuged again at 100,000 × *g* for 2 hours using a TLA-55 rotor (Optima MAX-MP, Beckman Coulter, Brea, CA, USA). The pellet was resuspended in PBS and stored at –80°C until further use.

### Collection and characterization of extracellular nanovesicles

ZH-1c-PEG2000-Chol-labeled BV2 cells (1 × 10^7^) were suspended in 1 mL of PBS and passed through 10 µm and 5 µm etched membranes (Whatman) at approximately 450 × *g* for 5 minutes, and the process was repeated five times for thorough purification. The supernatant was collected, centrifuged at 300 × *g* for 5 minutes to remove cell debris, and further clarified by centrifugation at 16,500 × *g* for 20 minutes. The resulting supernatant was filtered through a 0.22 µm membrane. BV2 cells were maintained in serum-free medium for 48 hours to collect conditioned medium. This medium underwent centrifugation at 16,500 × *g* for 20 minutes and filtration through a 0.22 µm filter. ENVs were pelleted by ultracentrifugation at 100,000 × *g* for 2 hours at 4°C and resuspended in 500 µL PBS for downstream characterization.

ZH-1c-ENVs morphology was examined using both cryogenic and conventional transmission electron microscopy (TEM). For negative staining, a 5 µL aliquot of ZH-1c-ENVs was deposited onto a copper grid, fixed, and imaged using an FEI Tecnai TEM (FEI, Hillsboro, OR, USA). Cryo-TEM imaging was performed by depositing 5 µL of sample onto a 200-mesh grid, vitrifying it rapidly in liquid ethane using an FEI Vitrobot (Thermo Fisher Scientific, Waltham, MA, USA), and acquiring images with an FEI Tecnai F20 system (Thermo Fisher Scientific). Morphology was further assessed using field-emission scanning electron microscopy (FESEM) (ZEISS, Oberkochen, Baden-Württemberg, Germany). Samples (5 µL) were placed on a 400-mesh grid, incubated at room temperature for 3 minutes, blotted, and stained with 1% uranyl acetate for 1 minute prior to imaging.

ZH-1c-ENVs and ENVs were lysed utilizing a Radio-Immunoprecipitation Assay (RIPA) buffer, and total protein concentrations were measured using a Micro-BCA Protein Assay Kit (Pierce, Thermo Fisher Scientific). Protein samples were analyzed via polyacrylamide gel electrophoresis and transferred onto polyvinylidene difluoride (PVDF) membranes. The membranes were blocked with PBS containing 5% skim milk and 0.05% Tween-20 at room temperature for 1 hour, then incubated overnight at 4°C with primary antibodies: anti-heat shock cognate 70 kDa protein (HSC70; rabbit polyclonal, 1:1000, Cell Signaling Technology, Danvers, MA, USA, Cat# 4872, RRID: AB_2279841), and anti-Annexin-2 (rabbit polyclonal, 1:1000, Abcam, Cambridge, UK, Cat# ab321903), anti-tumor susceptibility gene 101 protein (TSG101; rabbit monoclonal, 1:1000, Abcam, Cat# ab125011, RRID: AB_10974262), anti-CD59 (mouse monoclonal, 1:1000, Abcam, Cat# ab9183, RRID: AB_307054), anti-CD9 (rabbit monoclonal, 1:1000, Abcam, Cat# ab307085), and anti-CD55 (rabbit monoclonal, 1:1000, Abcam, Cat# ab133684, RRID: AB_2801387). Afterward, the membranes were incubated with HRP-conjugated secondary antibodies goat anti-rabbit IgG H&L (HRP) (1:5000, Abcam, Cat# ab6721, RRID: AB_955447) or HRP-conjugated secondary antibodies goat anti-mouse IgG H&L (HRP) (1:5000, Abcam, Cat# ab6789, RRID: AB_955439) at room temperature for 1 hour. Samples were washed three times with PBS containing 0.05% Tween-20, each for 10 minutes. Protein bands were visualized using an enhanced chemiluminescence (ECL) detection kit (SuperSignal™ West Pico PLUS Chemiluminescent Substrate, Thermo Fisher Scientific) and imaged with a ChemiDoc imaging system (Bio-Rad, Hercules, CA, USA).

A 10 µL aliquot of ZH-1c-ENVs or ENVs was applied to a microscope slide and incubated at 4°C for 1 hour. The slide was then blocked with 10 mg/mL bovine serum albumin for 20 minutes and washed with PBS. Custom-designed DNA probes complementary to the ZH-1c aptamer (GenScript, Nanjing, Jiangsu, China) were added and incubated in a humidified chamber at 37°C for 2 hours. After hybridization, the slides were washed with PBS and analyzed by fluorescence microscopy (Leica Microsystems, Wetzlar, Germany).

### Loading and release of dexmedetomidine

Dex (Sigma-Aldrich, Cat# D2895) was mixed with ZH-1c-ENVs or ENVs at a mass ratio of 1:10^6^. For ultrasound-assisted loading, the mixture was sonicated using a Model 505 dismembrator (0.25-inch tip) at 20% amplitude for six cycles (30 seconds on/off) with 2-minute intervals between cycles, followed by incubation at 37°C for 1 hour. Alternatively, passive loading was performed by stirring the Dex-vesicle mixture at room temperature for 1 hour. The prepared samples were subjected to particle size and zeta potential measurements by Zetasizer Nano ZS90 (Malvern Instruments, Worcestershire, UK). Unencapsulated Dex was removed using a Sephadex G25 chromatography column. Dex content in each group was quantified by high-performance liquid chromatography.

For release analysis, Dex-loaded ZH-1c-ENVs or ENVs were placed in a 300 kDa MWCO Float-A-Lyzer G2 dialysis device (Spectrum Laboratories, Rancho Dominguez, CA, USA) and incubated in PBS under gentle stirring at room temperature. Samples were collected at predefined time points and analyzed by high-performance liquid chromatography to determine the release profile.

### Fluorescent labeling of extracellular nanovesicles with fluorescein isothiocyanate

To assess cellular uptake and *in vivo* biodistribution, ZH-1c aptamer-modified ENVs were labeled with FITC (Sigma-Aldrich, Cat# F7250). A working solution was prepared by diluting 5 µL of FITC in 1 mL PBS. Equal volumes (1 mL each) of ENVs suspension and FITC solution were mixed and incubated in the dark at RT for 30 minutes. The reaction was terminated by adding 2 mL of 10% fetal bovine serum (FBS), followed by thorough mixing. Unbound FITC was removed by ultracentrifugation at 100,000 × *g* for 70 minutes, and the pellet was resuspended in PBS. The labeled ENVs were washed three times to eliminate residual free dye. Fluorescence intensity was evaluated using a fluorescence microscope (Leica Microsystems) and compared to unlabeled ENVs.

### Cell culture

BV2 cells were maintained in Dulbecco’s Modified Eagle Medium (DMEM; Gibco, Grand Island, NY, USA) enriched with 10% FBS (Gibco) and 1% penicillin–streptomycin solution (Gibco). Cultures were incubated under standard conditions of 37°C with 5% CO^2^ in a humidified atmosphere (Thermo Fisher Scientific). Upon reaching approximately 80% to 90% confluency, cells were subcultured and seeded into 6-well plates (Corning, Steuben County, NY, USA) at an initial density of 1 × 10^5^ cells/mL.

### Cell treatment and grouping

Aβ_1–42_ peptides (rPeptide; Beyotime, Beijing, China, Cat# P9001) were dissolved in hexafluoroisopropanol (Sigma-Aldrich) at 1 mg/mL and dried under a nitrogen stream. The resulting film was resuspended in DMSO and desalted into Tris–EDTA buffer using a 5 mL HiTrap desalting column (GE Healthcare, Chicago, IL, USA). Peptide concentration in the eluate was determined using the Bradford assay (Bio-Rad), followed by incubation at room temperature for 2 hours to promote oligomer formation. Prior to treatment, BV2 cells were cultured in serum-free DMEM and exposed to 5 μM Aβ_1–42_ oligomers or PBS for 48 hours.

Aβ_42_ peptides (Sigma-Aldrich, Cat# A9810) were dissolved in 100 mM NaOH to prepare a 2 mM stock solution, followed by 30 seconds of water bath sonication to obtain monomeric Aβ. The stock was then diluted with 10 mM HEPES, 100 mM NaCl, and 0.02% sodium azide to a final concentration of 25 μM, and incubated at room temperature for 21 days to induce fibril formation (Feng et al., 2024).

Cells were divided into six groups and cultured at identical densities: (1) Control: DMEM enriched with 10% FBS and 1% penicillin-streptomycin, without treatment. (2) Aβ_1–42_: Treated with 1 µg/mL Aβ_1–42_. (3) Aβ_1–42_ + Dex: Treated with 1 µg/mL Aβ_1–42_ and 10 µg/mL Dex. (4) Aβ_1__–42_ + ENVs@Dex: Treated with 1 µg/mL Aβ_1–42_ and 10 µg/mL Dex-loaded unmodified ENVs. (5) Aβ_1–42_ + ZH-1c-ENVs@Dex: Treated with 1 µg/mL Aβ_1–42_ and 10 µg/mL Dex-loaded, ZH-1c-modified ENVs. (6) Aβ_1–42_ + ZH-1c-ENVs@Dex + 3-TYP: Treated as in group 5 with the addition of 50 mM 3-TYP (Sirt3 inhibitor; HY-108331, MCE, Monmouth Junction, NJ, USA).

### Transmission electron microscopy

A 10 μL aliquot of Aβ oligomer or fibril solution prepared from Aβ_1–42_ peptide was applied to a 200-mesh carbon-coated copper grid and incubated at room temperature for 5 minutes to allow adsorption. Excess liquid was removed with filter paper, followed by negative staining with 10 μL of 2% phosphotungstic acid (pH 7.0) for 1 minute in the dark. After removing residual stain, the grid was air-dried and imaged using a TEM (HT7800, Hitachi, Tokyo, Japan; Liu et al., 2024).

### Analysis of cellular uptake of extracellular nanovesicles

To assess cellular uptake, FITC-labeled ENVs were added to BV2 cell culture medium at a concentration of 10 µg/mL and incubated for 24 hours. After incubation, cells were washed three times with PBS (pH 7.4; Gibco) to remove unbound ENVs. Intracellular fluorescence signals were visualized using a confocal laser scanning microscope (Leica Microsystems), and images were acquired to evaluate ENV’s internalization and distribution. For quantitative analysis, cells were harvested by trypsinization (trypsin-EDTA; Gibco), centrifuged at 300 × *g* for 5 minutes, resuspended in PBS, and passed through a 70 µm cell strainer (BD Biosciences, San Jose, CA, USA) to generate a single-cell suspension. Flow cytometry (FCM) was performed using a BD LSRFortessa analyzer (BD Biosciences) to determine the percentage of FITC-positive cells and mean fluorescence intensity. Appropriate negative controls were included to distinguish background fluorescence. FlowJo software (BD Biosciences) was employed for data processing and quantification of cellular uptake across experimental groups.

### Amyloid-beta uptake assay

FITC-labeled Aβ_1–42_ peptide (GenScript, Nanjing, China) was prepared as previously described (Chakrabarty et al., 2015). Briefly, Aβ_1–42_ was dissolved in 1% NH_4_OH (10 μL) and diluted with PBS to a final concentration of 1 mg/mL, followed by incubation at 37°C for 12 hours to induce oligomerization. The resulting solution was diluted to 5 µg/mL in DMEM/F-12 medium before application to BV2 cells. BV2 cells were pretreated with 40 ng/mL interleukin (IL)-4 and 100 ng/mL dexamethasone (Sigma-Aldrich, Cat# D4902) for 24 hours, then incubated with FITC-Aβ_1–42_ oligomers (5 µM) and serum antibodies (1 µM) in DMEM for another 24 hours. Cells were subsequently washed with PBS and fixed in 4% paraformaldehyde for 15 minutes at room temperature. After fixation, they were counterstained with DAPI in an antifade mounting medium for 5 minutes. Fluorescence imaging was performed using a CKX41-32PH automatic microscope (Olympus, Japan). The mean fluorescence intensity of Aβ_1–42_ oligomers per cell was quantified and normalized to control values, with at least 30 cells analyzed per group.

### Transwell blood–brain barrier model

To evaluate the transcytosis capability of ZH-1c-ENVs@Dex across the blood–brain barrier (BBB), a Transwell-based *in vitro* BBB model (Corning) was constructed using human brain microvascular endothelial cells (CRL-2299, ATCC). human brain microvascular endothelial cells were seeded onto 0.4 µm pore-size Transwell inserts at a density of 1 × 10^5^ cells/well in endothelial cell growth medium (PromoCell, Heidelberg, Germany) enriched with 10% FBS (Gibco) and 1% penicillin-streptomycin (Thermo Fisher Scientific). Cells were cultured at 37°C in a 5% CO_2_ incubator for 5–7 days until a confluent monolayer formed to simulate the BBB. FITC-labeled nanoparticles were diluted in medium to a final concentration of 100 µg/mL and added to the upper chamber. To monitor nanoparticle permeability, 200 µL of medium was collected from the lower chamber at 4-hour intervals and replaced with fresh medium. Fluorescence intensity was measured using a fluorescence spectrometer (Thermo Fisher Scientific) at excitation/emission wavelengths of 488 nm/525 nm. The accumulation of fluorescence in the lower chamber indicated nanoparticle transport efficiency across the BBB model (Qi et al., 2023).

### Proteomics sample preparation and measurement

Cells from the Aβ_1–42_ group and the Aβ_1–42_ + ZH-1c-ENVs@Dex group (*n* = 4) were washed twice with PBS to remove residual culture medium. The cells were then scraped into PBS and transferred to 1.5 mL or 2 mL centrifuge tubes. After centrifugation at 1000 × *g* for 5 minutes at 4°C, the supernatant was discarded. Cell pellets were lysed using an ultrasonic cell disruptor (SCIENTZ-IID, Scientz, Ningbo, China) in an ice bath with lysis buffer containing 10 mM DTT (Solarbio, Beijing, China, Cat# R0861), 1% protease inhibitor cocktail (Solarbio, Cat# P6731), 2 mM EDTA (Solarbio, Cat# E1170), and phenol extraction buffer (Merck, Rahway, NJ, USA, Cat# 100206). The disruption was performed for eight cycles. Equal volumes of Tris-saturated phenol (pH 8.0, BIOFOUNT, Beijing, China, Cat# HC1380) were added to the lysate, followed by vortexing for 4 minutes and centrifugation at 5000 × *g* for 10 minutes at 4°C. The upper phenol phase was collected and mixed with 0.1 M ammonium sulfate (Merck, Cat# 101217) and saturated methanol (Merck, Cat# 106035) at a 1:5 (v/v) ratio. The mixture was incubated overnight for protein precipitation. After centrifugation, the pellet was washed once with ice-cold methanol and three times with ice-cold acetone. The final protein pellet was dissolved in 8 M urea (Solarbio, Cat# U8020). Protein concentrations were quantified using a BCA protein assay kit (Beyotime, Cat# P0012).

### Proteolysis, peptide labeling, fractionation, and nano-LC-MS/MS analysis

Proteolysis was performed on 50 µg of protein per sample. Proteins were reduced with 5 mM DTT at 56°C for 30 minutes, followed by alkylation with 11 mM iodoacetamide in the dark at room temperature for 15 minutes. Samples were diluted to reduce urea concentration to < 2 M. Trypsin (Thermo Fisher Scientific, Cat# 25200056) was added at a 1:50 (w/w) enzyme-to-protein ratio for overnight digestion at 37°C, followed by a second digestion step using a 1:100 (w/w) ratio for an additional 4 hours.

Following trypsin digestion, peptides were desalted using HyperSep^TM^ C18 purification columns (Thermo Fisher Scientific, Cat# 60108-302) and vacuum-dried. The dried peptides were resuspended in 0.5 M TEAB (Thermo Fisher Scientific, Cat# 90114) and labeled with TMT reagents (Thermo Fisher Scientific, Cat# 90064CH). Each TMT reagent was dissolved in acetonitrile (Merck, Cat# 113212) and incubated with peptides for 2 hours at room temperature. After labeling, peptides were desalted, dried, and pooled in equal amounts. The combined sample was fractionated into 15 fractions using the Pierce^TM^ High pH Reversed-Phase Peptide Fractionation Kit (Thermo Fisher Scientific, Cat# 84868), then dried and reconstituted in 0.1% formic acid (Merck, Cat# 159002) for LC-MS/MS analysis.

For nano-LC-MS/MS, peptides (2 µg per fraction) were introduced into an Easy-nLC 1200 system (Thermo Fisher Scientific) via a C18 trapping column (100 µm × 20 mm, 5 µm particle size) and subsequently separated on an analytical C18 column (75 µm × 150 mm, 3 µm) at a constant flow rate of 300 nL/min. The mobile phase system consisted of solvent A (0.1% formic acid in water) and solvent B (0.1% formic acid in 95% acetonitrile). The gradient elution program was set as follows: 2%–8% B from 0–2 minutes, 8%–28% B from 2–71 minutes, 28%–40% B from 71–79 minutes, 40%–100% B from 79–81 minutes, and maintained at 100% B from 81–90 minutes. The eluates were analyzed on a Q-Exactive HFX mass spectrometer (Thermo Fisher Scientific) operating in positive ionization mode with a spray voltage of 2.1 kV. Full MS (MS1) spectra were acquired at a resolution of 60,000 (at m/z 200) over an m/z scan range of 350–1200, with an automatic gain control (AGC) target of 3 × 10^6^ and a maximum injection time of 30 ms. Data-dependent MS/MS (MS2) acquisition was carried out at a resolution of 15,000, AGC target set at 1 × 10^6^, and maximum injection time of 25 ms, using higher-energy collisional dissociation with a normalized collision energy of 32 and an isolation window of 2.0 Th.

### Data processing

Raw nano-LC-MS/MS data were processed using MaxQuant (v1.5.2.8) (https://www.maxquant.org/) for peptide identification and protein quantification. Spectra were searched against the UniProtKB Reference Proteome — Mus musculus (UP000000589, release 2024_03) (https://www.uniprot.org/proteomes/UP000000589), supplemented with a reverse decoy database. Trypsin/P was set as the cleavage enzyme, allowing up to two missed cleavages. The precursor mass tolerance was set to 20 ppm for the first search and 5 ppm for the main search; fragment mass tolerance was 0.02 Da. Peptide score distributions were also applied to enhance identification stringency. Differentially expressed proteins (DEPs) between the Aβ_1–42_ and Aβ_1–42_ + ZH-1c-ENVs@Dex groups were identified using the “limma” package in R (https://www.r-project.org/), with thresholds set at |log₂ fold change| > 0 and *P*-value < 0.05. Heatmaps of protein expression were generated using the R “heatmap” package, while volcano plots were created with the “ggplot2” package.

### Functional enrichment analysis

DEPs were analyzed for Gene Ontology (GO) and Kyoto Encyclopedia of Genes and Genomes (KEGG) pathway enrichment utilizing the clusterProfiler package in R. GO enrichment covered three functional categories: Biological Process (BP), Cellular Component (CC), and Molecular Function (MF). Bubble plots were generated using the enrichplot R package to display the enrichment analysis outcomes. KEGG pathway enrichment was also visualized in the same format to emphasize statistically significant signaling pathways.

### Measurement of Sirtuin 3 activity

Sirt3 activity was assessed using a fluorescence-based detection method. Total protein was extracted from BV2 cells and the hippocampal region of mouse brains using RIPA lysis buffer (Thermo Fisher Scientific). Protein concentration was determined with a BCA protein assay kit (Thermo Fisher Scientific). Sirt3 activity was then measured using the Sirt3 Activity Assay Kit (Abcam, Cat# ab156067). Briefly, total protein was incubated at room temperature for 1 hour with a reaction mixture containing Sirt3 assay buffer, a fluorescent substrate peptide, NAD, and a developer. Fluorescence intensity was measured at excitation/emission wavelengths of 350 nm/450 nm using a microplate fluorometer (Thermo Fisher Scientific, Cat# 23225).

### Cell Counting Kit-8 assay

The viability of cells in each group was assessed using a Cell Counting Kit-8 (CCK-8) kit (Beyotime, Cat# C0038). BV2 cells were cultured in DMEM (Gibco) enriched with 10% FBS (Gibco) and 1% penicillin-streptomycin (Gibco). Cells were seeded into 96-well plates at a density of 1 × 10^4^ cells per 100 μL per well and incubated for 24 hours. To evaluate the cytotoxicity of ZH-1c-ENVs@Dex on BV2 cells, ZH-1c-ENVs@Dex was added to the treatment group, while untreated cells served as the control group. After an additional 3-hour incubation, 10 μL of CCK-8 solution was added to each well, followed by a 2-hour incubation. Absorbance was monitored at 450 nm utilizing a microplate reader (Bio-Rad).

### Enzyme-linked immunosorbent assay

The concentrations of tumor necrosis factor-α (TNF-α; Cat# MTA00B), IL-6 (Cat# M6000B), IL-1β (Cat# MLB00C) (all from R&D Systems, Minneapolis, MN, USA), and Aβ_1–42_ (E-EL-M3010, Elabscience, Wuhan, China) in cell culture supernatants and hippocampal tissues were quantified utilizing enzyme-linked immunosorbent assay (ELISA) kits. BV2 cells were cultured in DMEM (Gibco) supplemented with 10% FBS and 1% penicillin-streptomycin (Gibco), seeded into 6-well plates at 5 × 10^5^ cells/mL, and incubated for 24 hours at 37°C with 5% CO_2_. Supernatants were collected and centrifuged at 1500 × *g* for 10 minutes to remove debris. For tissue analysis, hippocampal samples were homogenized in cold PBS and processed identically.

Samples and standards were added in triplicate to microplate wells and incubated for 2 hours at room temperature. After discarding the supernatant and washing, detection antibodies were added and incubated for 1 hour, followed by enzyme conjugates for 30 minutes. Colorimetric development was performed with TMB substrate for 15 minutes and stopped with 2N H_2_SO_4_. Absorbance was measured at 450 nm using a microplate reader (Bio-Rad), and cytokine levels were calculated based on standard curves.

### Apoptosis detection

Apoptosis in BV2 cells was assessed using an Annexin V-FITC/PI dual-staining kit (BD Biosciences, Cat# 556547). Cells were harvested with 0.25% trypsin (Gibco), centrifuged at 300 × *g* for 5 minutes, and washed twice with PBS. The resulting cell pellets were resuspended in 1× Binding Buffer at a density of 1 × 10^6^ cells/mL. Subsequently, 100 µL of the cell suspension from each group was mixed with 5 µL Annexin V-FITC and 5 µL PI dye, gently vortexed, and incubated in the dark at room temperature for 15 minutes. After incubation, the stained cell suspension was transferred to FCM tubes containing 400 µL of 1× Binding Buffer, mixed thoroughly, and immediately analyzed. FCM was conducted using a BD FACSCalibur instrument (BD Biosciences) with 488 nm excitation, and the fluorescence signals of FITC and PI were detected. Data were analyzed with FlowJo software to differentiate cell populations, including early apoptotic cells (Annexin V^+^/ PI^–^), late apoptotic cells (Annexin V^+^/ PI^+^), and necrotic cells (Annexin V^–^/PI^+^), based on Annexin V and PI fluorescence signals.

### Lysosomal activity assay

To evaluate the effect of ZH-1c-ENVs@Dex on lysosomal activity in BV2 cells, the LysoSensor Green probe (L7535, Thermo Fisher Scientific) was used to stain the cells in each group. BV2 cells were maintained in DMEM (Gibco) supplemented with 10% FBS and 1% penicillin-streptomycin (Thermo Fisher Scientific) at 37°C in a humidified 5% CO_2_ incubator until reaching 70%–80% confluence. Cells were then incubated with 1 μM LysoSensor Green at 37°C for 30 minutes in the dark. After staining, cells were washed three times with PBS to remove excess dye. Lysosomal fluorescence was examined using a fluorescence microscope (Leica Microsystems) at an excitation/emission wavelength of 488 nm/525 nm. The intensity and distribution of green fluorescence were used as indicators of lysosomal activity.

### Lysosomal colocalization assay

To evaluate the direct interaction between ZH-1c-ENVs@Dex and lysosomes, a lysosomal colocalization assay was performed. First, ZH-1c-ENVs@Dex was fluorescently labeled using a FITC labeling kit (Thermo Fisher Scientific, Cat# C10237). The labeled ZH-1c-ENVs@Dex were added to the culture medium of BV2 cells and incubated at 37°C for 4 hours to observe their intracellular distribution. For *in vivo* analysis, FITC-labeled ZH-1c-ENVs@Dex (20 mg/kg) were administered via tail vein injection into six 8-week-old male Sprague–Dawley rats (220–250 g), which were obtained from Charles River Laboratories (Beijing, China; license No. SCXK (Jing) 2016-0006) and equally divided into control and experimental groups (*n* = 3 per group). All animals were maintained under specific-pathogen-free (SPF) conditions in a controlled environment with a 12-hour light/dark cycle, ambient temperature of 22 ± 2°C, and relative humidity of 55% ± 10%. Rats were housed in groups of 2–3 per cage with free access to food and water. Throughout the surgical procedures, anesthesia was induced and maintained via inhalation of 2%–3% isoflurane (Piramal Critical Care, Bethlehem, PA, USA). After 24 hours, brain and major organs were harvested for tissue analysis. For both cells and tissue sections, fixation was performed with 4% paraformaldehyde (Sigma-Aldrich) for 20 minutes, followed by permeabilization with 0.1% Triton X-100 (Sigma-Aldrich) for 10 minutes and blocking with 5% bovine serum albumin (Beyotime) for 1 hour. Frozen tissue sections (30 μm) were prepared accordingly. Samples were then incubated overnight at 4°C with primary antibodies against Rab5 (1:500, Cat# 3547S, RRID: AB_2300649, Cell Signaling Technology) and Lamp1 (1:100, Cat# ab320851, Abcam). After washing, Alexa Fluor 488-conjugated secondary antibodies (1:1000, Invitrogen, Carlsbad, CA, USA, Cat# A-11034, RRID: AB_2576217) were applied for 1 hour at room temperature in the dark. Nuclei were counterstained with DAPI (Beyotime) for 10 minutes. Colocalization was assessed under a fluorescence microscope (Leica Microsystems).

### Live/dead fluorescence staining

To evaluate the biocompatibility of ZH-1c-ENVs@Dex with BV2 cells, a Live/Dead fluorescence staining assay was conducted. BV2 cells were seeded in 96-well plates at 1 × 10^4^ cells/well and incubated for 24 hours, followed by treatment with ZH-1c-ENVs@Dex for another 24 hours. Cell viability was assessed using a Calcein AM/PI dual staining kit (Invitrogen, Cat# L3224). The cell viability and mortality were then visualized and recorded using a fluorescence microscope (Leica Microsystems).

### Alzheimer’s disease mouse model and grouping

A total of 72 mice were used in this study (12 per group), including seven-month-old female APP/PS1 transgenic AD mice (*n* = 60, 26 ± 2 g) and age-matched C57BL/6J wild-type controls (*n* = 12, 26 ± 2 g). They were obtained from Beijing HFK Bioscience Co., Ltd. All animals were maintained under SPF conditions and housed six mouse per cage in IVCs, with ad libitum access to food and water. Housing conditions included a 12-hour light/dark cycle, a stable temperature of 22 ± 2°C, and 55% ± 10% relative humidity. Finally, mice were anesthetized using 2%–3% isoflurane for further experiments.

Animals were randomly assigned to six groups: WT group (healthy C57BL/6J mice), AD group (APP/PS1 transgenic AD mice), AD + Dex group (APP/PS1 transgenic AD mice receiving tail vein injections of free Dex at 20 μg/kg), AD + ENVs@Dex group (APP/PS1 transgenic AD mice receiving tail vein injections of unmodified ENVs loaded with Dex at 25 µg/mL), AD + ZH-1c-ENVs@Dex group (APP/PS1 transgenic AD mice receiving tail vein injections of ZH-1c aptamer-modified ENVs loaded with Dex at 25 µg/mL), and AD + ZH-1c-ENVs@Dex + 3-TYP group (APP/PS1 transgenic AD mice receiving tail vein injections of ZH-1c aptamer-modified ENVs loaded with Dex at 25 µg/mL and the Sirt3 inhibitor 3-TYP at 50 mg/kg). Body weight and behavioral parameters were monitored daily (Ruiz and Cobra, 1966; Qiao et al., 2023).

### Behavioral experiments

To evaluate the learning and memory abilities of the mice in each group, the Morris water maze (MWM) test (Vorhees and Williams, 2006) was conducted. Mice were trained to locate a submerged platform in an opaque pool across five consecutive days, with four trials per day. Escape latency was recorded for each trial. On day 6, the platform was removed, and a probe trial was conducted to track swimming trajectories and assess memory retention. To evaluate sustained efficacy, the MWM was repeated 6 weeks after treatment.

### Fluorescence immunostaining

Hippocampal tissues from mice in each group were collected and prepared as frozen sections. Cells or brain tissue sections were fixed on slides using 4% paraformaldehyde (Sigma-Aldrich) for 20 minutes and permeabilized with 0.1% Triton X-100 (Sigma-Aldrich) for 10 minutes. Subsequently, the samples were blocked with 5% bovine serum albumin (Beyotime) for 1 hour at room temperature. Primary antibodies, including anti-ionized calcium-binding adapter molecule 1 (Iba1; 1:500, Wako, Osaka, Japan, Cat# 019-19741, RRID: AB_839504), anti-Lamp1 (1:100, Abcam, Cat# ab320851), and anti-Aβ_1–42_ (1:500, Proteintech, Rosemont, IL, USA, Cat# 60342-1-Ig, RRID: AB_2881451), were incubated with the sections overnight at 4°C. The next day, sections were incubated with Alexa Fluor 488-conjugated secondary antibody (Invitrogen, Cat# A-11034, 1:1000, RRID: AB_2576217) and Alexa Fluor 647-conjugated secondary antibody (Abcam, Cat# ab150115, 1:1000, RRID: AB_2732856) for 1 hour at room temperature in the dark. Nuclei were counterstained with DAPI (C1005, Beyotime) for 10 minutes. Stained samples were imaged using a fluorescence microscope (Leica Microsystems).

### Hematological and biochemical blood analysis

Mouse tail vein blood samples were collected weekly using EDTA-coated anticoagulant tubes (BD, Franklin Lakes, NJ, USA). Hematological parameters, including white blood cell, red blood cell, and platelet counts, were measured using an automated hematology analyzer (Sysmex, Kobe, Japan). Serum levels of alanine aminotransferase, aspartate aminotransferase, blood urea nitrogen, and creatinine were determined with an automatic biochemical analyzer (Hitachi) to assess liver and kidney function.

### Histopathological analysis

At the end of the study, mice were euthanized using intraperitoneal injection of 1% pentobarbital sodium (50 mg/kg; Sigma-Aldrich) and major organs (liver, kidneys, spleen, heart, and lungs) were harvested and fixed in 10% neutral buffered formalin (Sigma-Aldrich). Tissues were embedded in paraffin, sectioned, and stained with hematoxylin and eosin (H&E; Thermo Fisher Scientific). Morphological changes were examined under a light microscope (Olympus, Tokyo, Japan), and representative images were captured for analysis.

### *In vivo* biodistribution

To evaluate the distribution of ZH-1c-ENVs@Dex in AD mice, an *in vivo* imaging system (PerkinElmer, Waltham, MA, USA) was employed. AD mice were randomly divided into three groups (*n* = 3 per group): Control, fluorescently labeled ENVs@Dex, and ZH-1c-ENVs@Dex. Both ENVs@Dex and ZH-1c-ENVs@Dex were fluorescently labeled using an FITC labeling kit (Thermo Fisher Scientific). The labeled ENVs were administered via tail vein injection at a dose of 20 mg/kg.

Fluorescence imaging was conducted at 8, 16, and 24 hours post-injection. Mice were anesthetized with 2%–3% isoflurane and positioned in the *in vivo* imaging system chamber for whole-body imaging. Fluorescence intensity at each time point was recorded to monitor the *in vivo* distribution and retention of ENVs. At 48 hours, animals were euthanized, and major organs (brain, heart, liver, spleen, lungs, and kidneys) were harvested, rinsed in PBS, and subjected to *ex vivo* imaging using the same system. Following imaging, organs were immediately placed in ice-cold PBS and processed for cryosectioning. Frozen sections (10 μm thick) were prepared using a cryostat (Leica CM1950, Leica Microsystems), mounted onto glass slides, and stained with DAPI (Sigma-Aldrich) for nuclear visualization. Fluorescence distribution within tissues was observed using a fluorescence microscope (Leica DMi8, Leica Microsystems), and signal intensities were quantified using ImageJ software (version 1.53t, National Institutes of Health, Bethesda, MD, USA). Regions of interest were selected manually, and the background fluorescence was subtracted before analysis. All measurements were performed under identical imaging settings to ensure comparability. This comprehensive analysis enabled a precise assessment of the organ-level accumulation and biodistribution of ENVs.

### Immunohistochemistry staining

To examine the activation status of BV2 cells and the expression of neuroinflammatory markers in brain tissue, immunohistochemistry staining was performed. Brain tissues were initially fixed in 4% paraformaldehyde (Sigma-Aldrich), followed by paraffin embedding and sectioning. Immunostaining was performed with primary rabbit anti-Iba1 antibody (1:500; Abcam, Cat# ab178846, RRID: AB_2892453), followed by secondary biotinylated goat anti-rabbit IgG (H+L) antibody (1:200; Abcam, Cat# ab6720, RRID: AB_955447). Images were captured using a Nikon microscope (Nikon, Tokyo, Japan) for further analysis.

### Nissl staining

To assess neuronal survival in the hippocampus of AD mice, Nissl staining was conducted. Brain tissues were harvested and fixed in 10% neutral buffered formalin (Sigma-Aldrich) for 24 hours. Coronal sections (30 μm thick) encompassing the hippocampal region were prepared. Sections were incubated in 0.1% Nissl staining solution (Beyotime) at 37°C for 2 hours. After staining, sections were rinsed with PBS, sequentially dehydrated with graded ethanol (70%, 95%, and 100%), and cleared in xylene. Stained neurons were observed and imaged under a light microscope (Nikon).

### Quantitative reverse transcription-polymerase chain reaction

Total RNA was extracted from cells using Trizol reagent (Invitrogen). BV2 cells (ATCC) were cultured in DMEM enriched with 10% FBS and 1% penicillin-streptomycin. Cells were seeded in 6-well plates at a density of 5 × 10^5^ cells/mL and incubated at 37°C in a humidified incubator with 5% CO_2_ for 24 hours. RNA concentration and purity were assessed using a NanoDrop 2000 spectrophotometer (Thermo Fisher Scientific), ensuring an A260/A280 ratio between 1.8 and 2.0. Reverse transcription was performed using the PrimeScript RT reagent kit (Takara, Kusatsu, Shiga, Japan) in a 20 µL reaction system containing 1 µg RNA, 4 µL 5X PrimeScript buffer, 1 µL PrimeScript RT enzyme, 1 µL random primers, and RNase-free water. Quantitative reverse transcription-polymerase chain reaction (qRT-PCR) was conducted on an ABI 7500 Fast Real-Time PCR System (Applied Biosystems, Foster City, CA, USA) using SYBR Green PCR Master Mix (Takara). Each 20 µL reaction mixture consisted of 10 µL SYBR Green Master Mix, 0.8 µL forward primer (10 µM), 0.8 µL reverse primer (10 µM), 2 µL cDNA, and 6.4 µL nuclease-free water. Thermal cycling conditions included an initial denaturation at 95°C for 30 seconds, followed by 40 cycles of 95°C for 5 seconds and 60°C for 34 seconds.

The primer sequences utilized for qRT-PCR were as follows: nuclear factor kappa B (*NF-κB*), forward 5′-ACA ACT ATG AGA TGA ACTC CGG G-3′ and reverse 5′-GAT AGC AGT GGG CTG TCT CC-3′; inducible nitric oxide synthase (*iNOS*), forward 5′-GGT AGA GGC CTG GAA AAC CC-3′ and reverse 5′-ATG GTG ACT CTG ACT CGG GA-3′; *COX-2*, forward 5′-GCG ACA TAC TCA AGC AGG AGC A-3′ and reverse 5′-AGT GGT AAC CGC TCA GGT GTT G-3′; glyceraldehyde-3-phosphate dehydrogenase (*GAPDH*), forward 5′-CAT CAC TGC CAC CCA GAA GAC TG-3′ and reverse 5′-ATG CCA GTG AGC TTC CCG TTC AG-3′. Gene expression levels were quantified utilizing the 2^–ΔΔCt^ method, with *GAPDH* serving as the internal reference gene. All experiments were performed in triplicate, and the mean value of each group was used for analysis.

### Western blotting

Total protein was extracted from BV2 cells and the hippocampal region of mouse brains using RIPA lysis buffer (Thermo Fisher Scientific). BV2 cells were maintained in DMEM/F-12 medium enriched with 10% FBS and 1% penicillin-streptomycin for 24 hours prior to lysis. Protein concentrations were determined using a BCA assay kit (Thermo Fisher Scientific). Equal amounts of protein samples were separated by SDS-PAGE and transferred onto PVDF membranes (Millipore, Burlington, MA, USA). The membranes were blocked with 5% non-fat milk for 1 hour and then incubated overnight at 4°C with the following primary antibodies: NF-κB (1:1000, Cell Signaling Technology, Cat# 8242S, AB_10859369) and COX-2 (1:1000, Cell Signaling Technology, Cat# 12282S, RRID: AB_2798010), iNOS (1:1000, Abcam, Cat# ab178945, RRID: AB_2734514), Lamp1 (1:1000, Abcam, Cat# ab24170, RRID: AB_775978), cathepsin B (CatB; 1:1000, Abcam, Cat# ab227811, RRID: AB_2890731), V-ATPase (1:2000, Abcam, Cat# ab199326, RRID: AB_2889856), and Sirt3 (1:1000, Abcam, Cat# ab246522, RRID: AB_2923807). GAPDH (1:1000, Abcam, Cat# ab8245, RRID: AB_2107448) was used as the internal control. The membranes were then incubated with HRP-conjugated secondary antibodies Goat anti-rabbit IgG H&L (HRP) (1:5000, Abcam, Cat# ab6721). Protein bands were detected utilizing ECL reagents (Thermo Fisher Scientific) and visualized with the ChemiDoc imaging system (Bio-Rad). Densitometric analysis of band intensities was performed using Image Lab software (Bio-Rad).

### Statistical analysis

Statistical evaluations were carried out using GraphPad Prism version 9.5.0 (GraphPad Software, San Diego, CA, USA, www.graphpad.com). Data are presented as the mean ± standard deviation (SD). For comparisons involving two groups, unpaired Student’s *t*-tests were applied. In cases involving more than two groups, one-way analysis of variance followed by Tukey’s multiple comparisons test was utilized. A *P*-value of < 0.05 was considered statistically significant.

## Results

### Preparation and characterization of ZH-1c-ENVs

**Figure 1:**
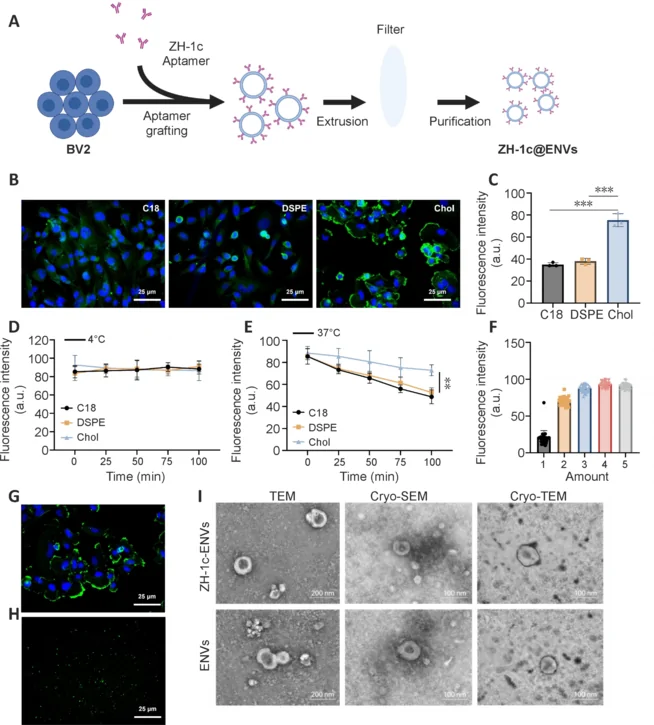
Preparation and characterization of ZH-1c-ENVs. (A) Schematic illustration of the preparation of ZH-1c-ENVs. (B) Fluorescent confocal microscopy images showing the labeling efficiency of FITC-labeled C18-PEG_2000_, DSPE-PEG_2000_, and cholesterol-PEG_2000_ on cell membranes, scale bars: 25 μm. (C) Quantitative analysis of fluorescence intensity for different lipid molecules on cell membranes. (D) Fluorescence intensity retention of different lipid molecules at 4°C. (E) Fluorescence intensity retention of different lipid molecules at 37°C. (F) MALDI-TOF-MS analysis of the synthesis of ZH-1c-PEG_2000_-chol. (G) Fluorescent images of FITC-labeled ZH-1c-PEG_2000_-chol on microglial cell membranes. Scale bar: 25 μm. (H) Fluorescent images of ZH-1c-ENVs. Scale bar: 25 μm. (I) Morphological characterization of ZH-1c-ENVs by transmission electron microscopy. Scale bars: 200 nm. Cellular experiments were conducted in triplicate. Data in C–F are presented as mean ± SD (*n* = 3 independent experiments). **P < 0.01, ***P < 0.001. C18: Stearoyl; Chol: cholesterol; DSPE: distearoylphosphatidylethanolamine; ENVs: extracellular nanovesicles; FITC: fluorescein isothiocyanate; SEM: scanning electron microscopy; TEM: transmission electron microscopy.

To facilitate the anchoring of lipid-conjugated aptamers onto cell membranes for the preparation of aptamer-modified ENVs (**Figure 1A**), we optimized the lipid type and dosage. FITC-labeled C18-PEG_2000_, DSPE-PEG_2000_, and cholesterol-PEG_2000_ were evaluated. Confocal fluorescence microscopy showed that all three lipids readily inserted into the cell membrane, but with varying labeling efficiencies (**Figure 1B**). Quantitative analysis revealed that chol-PEG_2000_ produced the highest average fluorescence intensity among the tested lipids (**Figure 1C**). This superior labeling efficiency is likely due to the physicochemical characteristics of cholesterol. While C18-PEG_2000_ and DSPE-PEG_2000_ possess long hydrophobic tails and hydrophilic PEG heads that favor micelle or liposome formation, this self-assembly can impair their incorporation into the membrane. In contrast, the amphiphilic and rigid structure of cholesterol allows chol-PEG_2000_ to insert more effectively into the lipid bilayer. Additionally, cholesterol enhances hydrophobic interactions within the bilayer, contributing to increased membrane rigidity and ENV stability (Moghimi and Patel, 1988).

The stability of lipid-conjugated aptamers on cell surfaces is crucial for the successful generation of ZH-1c-ENVs. To evaluate this stability, we monitored fluorescence decay over time at 4°C and 37°C to evaluate the retention of C18-PEG_2000_, DSPE-PEG_2000_, and chol-PEG_2000_. At 4°C, all three lipids exhibited a gradual decrease in fluorescence intensity to approximately 85% within 100 minutes, indicating strong membrane retention under low-temperature conditions (**Figure 1D**). In contrast, at 37°C, fluorescence intensity for the same lipids declined more rapidly, reaching about 50% within the same timeframe. Among the tested lipids, chol-PEG_2000_ demonstrated stronger fluorescence intensity compared to C18-PEG_2000_ and DSPE-PEG_2000_ at 37°C (**Figure 1E**). Considering both fluorescence labeling efficiency and signal stability, chol-PEG_2000_ was selected for subsequent experiments.

We next optimized the dosage of chol-PEG_2000_ for membrane labeling. Suboptimal doses may result in insufficient surface conjugation, while excessive amounts can lead to membrane blebbing and lipid bilayer disruption. Fluorescence intensity increased with increasing doses of chol-PEG_2000_ from 1 to 4 nmol (**Figure 1F**). To evaluate the labeling efficiency, 1–5 µL of 1 mM chol-PEG_2000_ was added to 250 µL of the cell suspension (**Additional Figure 1A**), and the fluorescence intensity of FITC-labeled chol-PEG_2000_ was compared before and after labeling across different doses (**Additional Figure 1B**). The amount of lipid incorporated into the membrane increased in a dose-dependent manner (**Additional Figure 1C**). The labeling efficiency, defined as the fraction of fluorescence intensity change relative to the initial FITC-labeled chol-PEG_2000_, was 14.73%, 16.64%, 18.65%, 24.81%, and 20.56% for doses of 1, 2, 3, 4, and 5 nmol, respectively. Based on these results, 4 nmol was determined to be the optimal concentration for effective and stable membrane labeling.

We synthesized ZH-1c-PEG_2000_-chol and isolated microglial cells, which were cultured and expanded *in vitro* for 5 days (**Additional Figure 1D**). Using an optimized labeling protocol, we confirmed that FITC-labeled ZH-1c-PEG_2000_-chol was successfully anchored to the microglial cell membrane (**Figure 1G**). Following labeling, approximately 107 microglial cells (purity: **~**90%, characterized by CD11c, **Additional Figure 1E**) were processed through an extruder to produce ZH-1c-ENVs. Super-resolution structured illumination microscopy (SIM) revealed that ZH-1c-ENVs displayed diameters ranging from 110 to 300 nm (**Figure 1H**). Nanoparticle tracking analysis showed a size distribution of 60–250 nm, with a peak at 100 nm. In contrast, ENVs derived from unlabeled microglial cells ranged from 30 to 220 nm, peaking at 90 nm. Electron microscopy demonstrated that both ZH-1c-ENVs and control ENVs possessed a characteristic disc-like morphology (**Figure 1I**). Cryo-transmission and cryo-scanning electron microscopy images further confirmed that both types of vesicles were spherical and enclosed by a lipid bilayer.

Western blot analysis identified cytoplasmic proteins Annexin II, TSG101, and HSC70, as well as membrane-associated proteins CD59, CD9, and CD55 in both ZH-1c-ENVs and ENVs (**Additional Figure 2A**). These findings suggest that these proteins are encapsulated within ZH-1c-ENVs during the extrusion process. CD55 and CD59 confer resistance to complement-mediated lysis, enhancing vesicle stability in circulation, while CD9 facilitates membrane fusion and targeted delivery. Stability assessment by monitoring particle size at peak distribution revealed that both ZH-1c-ENVs and ENVs (**~**100 nm) maintained structural integrity with no significant aggregation or degradation when stored in PBS at –80°C for up to 6 months (**Additional Figure 2B**). These results confirm that ZH-1c-ENVs can be efficiently produced at scale, stably cryopreserved, and reconstituted for downstream applications without loss of quality.

We further confirmed the presence of ZH-1c on conjugated ENVs using Cy5-labeled ZH-1c hybrid probes (h-probes) and random probes as controls (c-probes). Fluorescence intensity analysis showed strong red signals exclusively in dot blots of ZH-1c-ENVs incubated with h-probes, whereas the other three groups exhibited only weak signals, likely attributable to minimal nonspecific probe retention (**Additional Figure 2C**). To assess targeting specificity, approximately 8 × 10^5^ ZH-1c-ENVs or control ENVs were incubated with microglial cells expressing membrane CD64. After just 10 minutes, ZH-1c-ENVs demonstrated robust binding to microglial surfaces, while PKH67-labeled control ENVs exhibited minimal interaction. At 30 minutes, control ENVs showed limited adhesion and fusion, whereas ZH-1c-ENVs displayed significantly stronger fluorescence intensity, indicating enhanced cellular association (**Additional Figure 2D** and **E**).

Collectively, these results confirm that ZH-1c-ENVs rapidly and specifically bind to nucleophosmin on microglial cell surfaces, enabling efficient and targeted delivery.

### Preparation and characterization of ZH-1c-ENVs@Dex

**Figure 2:**
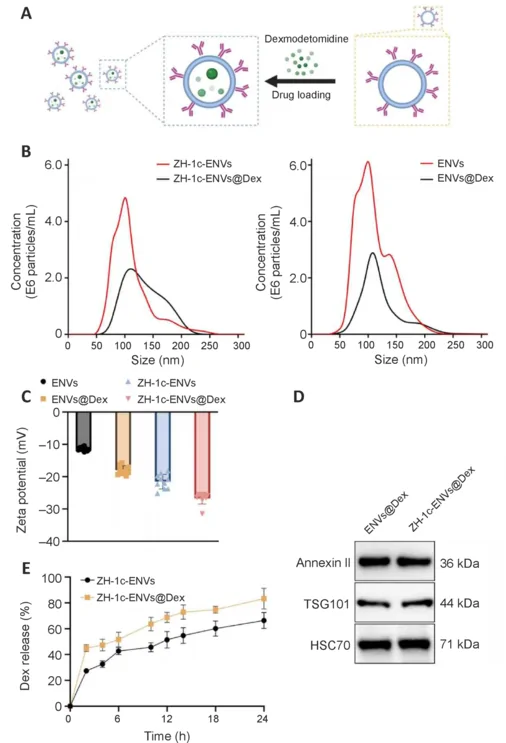
Preparation and Characterization of ZH-1c-ENVs@Dex and ENVs. (A) Schematic illustration of Dex loading into ZH-1c-ENVs via ultrasonication at room temperature. (B) Particle size distribution of ZH-1c-ENVs and ENVs before and after Dex loading. (C) Zeta potential changes in ZH-1c-ENVs and ENVs before and after Dex loading. (D) Western blot analysis of Annexin II, TSG101, and HSC70 protein expression in ZH-1c-ENVs and ENVs after Dex loading. (E) Release kinetics of Dex from ZH-1c-ENVs and ENVs at 37°C. Experiments were conducted in triplicate. Data in C, E are presented as mean ± SD (*n* = 3 independent experiments). Dex: Dexmedetomidine; ENVs: extracellular nanovesicles; HSC70: heat shock cognate 70 kDa protein; TSG101: tumor susceptibility gene 101 protein.

Dex was loaded into ZH-1c-ENVs and ENVs using either ultrasonic treatment or passive incubation at ambient temperature (**Figure 2A**). Ultrasonic loading yielded significantly higher efficiency, with encapsulation rates of 23.56% ± 2.13% for ZH-1c-ENVs and 27.6% ± 1.89% for ENVs. In contrast, passive incubation through mixing and stirring achieved considerably lower efficiencies, at 4.02% ± 1.22% for ZH-1c-ENVs and 7.56% ± 2.23% for ENVs. The enhanced loading observed with ultrasonication can be attributed to transient reductions in membrane microviscosity, which facilitate drug penetration. Membrane integrity was rapidly restored post-sonication, and no significant alterations were detected in membrane lipid composition or associated surface proteins. Therefore, ultrasonic treatment was selected as the preferred method for Dex encapsulation in all subsequent experiments. After Dex loading, the overall particle size distribution shifted slightly to the right, with the sizes of ZH-1c-ENVs and ENVs increasing to 110 nm and 108 nm, respectively, at peak concentration (**Figure 2B**). Additionally, the average zeta potential of both ZH-1c-ENVs and ENVs decreased after Dex loading (**Figure 2C**). Western blot analysis showed a marked reduction in the cytoplasmic proteins Annexin II, TSG101, and HSC70, indicating that these proteins were largely lost during ultrasonication. However, membrane proteins remained intact, suggesting that the targeting capacity of the vesicles was not significantly compromised (**Figure 2D**).

The release kinetics of Dex from ZH-1c-ENVs and ENVs were evaluated at 37°C. Both nanocarriers exhibited an initial burst release within the first hour, followed by a sustained-release phase. After 24 hours, cumulative release reached approximately 68.56% from ZH-1c-ENVs and 78.15% from ENVs (**Figure 2E**). This biphasic release profile indicates that Dex-loaded nanovesicles enable both immediate and prolonged drug delivery, providing potential advantages for targeted therapeutic applications.

### ZH-1c-ENVs@Dex demonstrates enhanced microglial uptake and blood–brain barrier penetration

**Figure 3:**
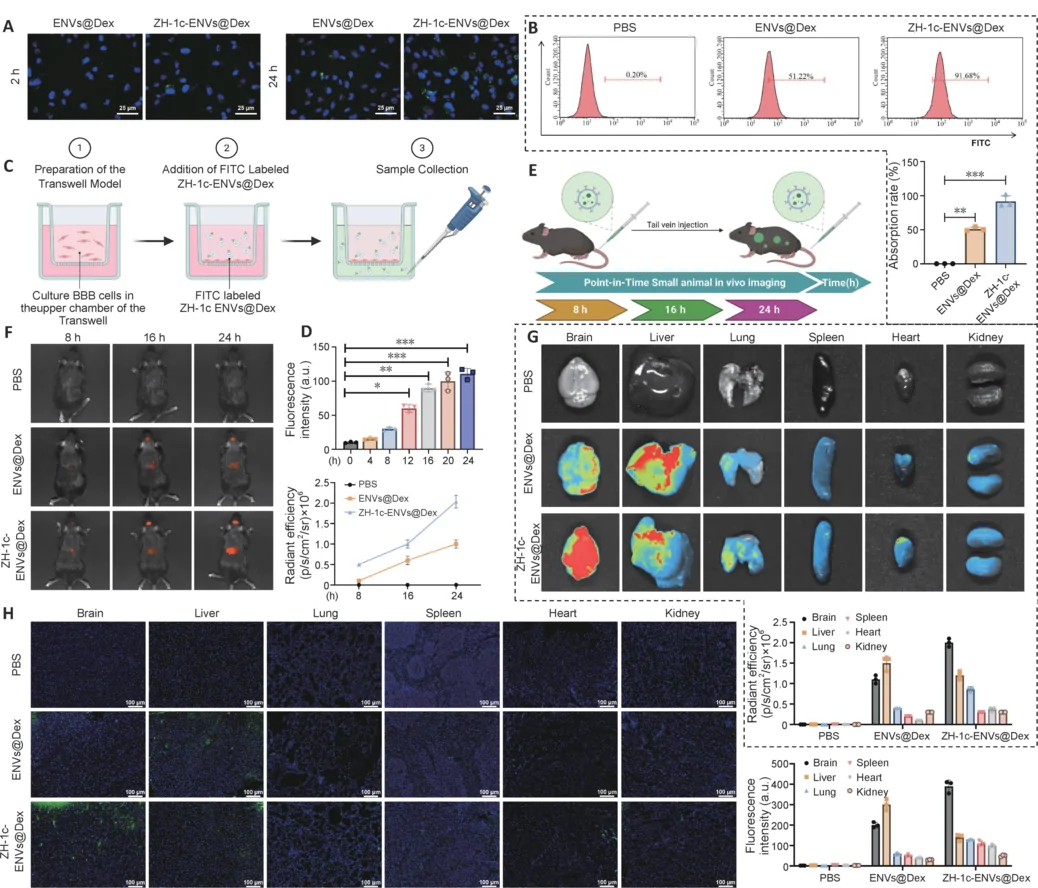
Uptake of ZH-1c-ENVs@Dex by BV2 microglial cells and BBB penetration capability. (A) Uptake of ZH-1c-ENVs@Dex and ENVs@Dex by BV2 microglial cells observed under confocal microscopy, with fluorescence intensity comparison; scale bars: 25 μm. (B) Quantitative analysis of ZH-1c-ENVs@Dex and ENVs@Dex uptake rates in BV2 microglial cells by flow cytometry. (C) Schematic representation of the Transwell BBB model. (D) Changes in fluorescence intensity of ZH-1c-ENVs@Dex crossing the BBB model, with quantitative analysis of fluorescence in the lower chamber at different time points. (E) Distribution of fluorescently labeled ZH-1c-ENVs@Dex in mice following tail vein injection, monitored using an *in vivo* imaging system. (F) Fluorescence intensity in the mouse brain at different time points post-injection, as measured by *in vivo* imaging system. (G) Fluorescence imaging of excised mouse brain and major organs 24 hours post-tail vein injection, comparing the distribution of ZH-1c-ENVs@Dex and unmodified ENVs. (H) Tissue sections of major organs and fluorescence microscopy images further validate the distribution of ZH-1c-ENVs@Dex in the brain and other organs; scale bars: 100 μm. Data in B, D, H are presented as mean ± SD. Cellular experiments were conducted in triplicate (*n* = 3). *P < 0.05, **P < 0.01, ***P < 0.001. BBB: Blood–brain barrier; Dex: dexmedetomidine; ENVs: extracellular nanovesicles; PBS: phosphate-buffered saline.

To assess cellular uptake by microglia, ZH-1c-ENVs@Dex and ENVs@Dex were fluorescently labeled and incubated with BV2 microglial cells. Confocal microscopy revealed a markedly stronger intracellular FITC signal in cells treated with ZH-1c-ENVs@Dex than those treated with unmodified ENVs@Dex, suggesting that aptamer functionalization substantially enhanced nanoparticle internalization (**Figure 3A**). FCM quantitatively confirmed this observation, showing that ZH-1c-ENVs@Dex achieved an uptake rate of 88.7%, significantly surpassing the 43.6% uptake observed for ENVs@Dex (**Figure 3B**). Collectively, these results demonstrate that aptamer modification significantly improves the uptake of ENVs by BV2 microglial cells.

To further evaluate the ability of ZH-1c-ENVs@Dex to traverse the BBB, a Transwell BBB model was employed, as illustrated in **Figure 3C**. FITC-labeled nanoparticles were added to the apical compartment, and fluorescence intensity in the basolateral chamber was monitored over time. A time-dependent increase in fluorescence intensity was detected within 24 hours, indicating effective translocation of nanoparticles across the BBB model (**Figure 3D**). These findings demonstrate that ZH-1c-ENVs@Dex possesses excellent BBB penetration capabilities, enabling efficient delivery to the brain.

Additionally, fluorescently labeled ZH-1c-ENVs@Dex were administered via tail vein injection, and their biodistribution in mice was monitored using an *in vivo* imaging system (**Figure 3E**). The results showed that ZH-1c-ENVs@Dex predominantly accumulated in the brain, with fluorescence intensity peaking at 4 hours post-injection and remaining high even at 24 hours (**Figure 3F**). Quantitative fluorescence analysis, shown in the right panel of **Figure 3F**, confirmed sustained cerebral retention over time. *Ex vivo* imaging of major organs at 24 hours post-administration revealed pronounced fluorescence accumulation in the brain for ZH-1c-ENVs@Dex, whereas ENVs predominantly localized to the liver (**Figure 3G**). Further tissue sectioning and fluorescence microscopy of major organs corroborated the *ex vivo* imaging findings (**Figure 3H**). These results suggest that ZH-1c aptamer modification not only enhances the distribution of ZH-1c-ENVs@Dex to the brain but also prolongs their retention time *in vivo*.

### ZH-1c-ENVs@Dex exhibits excellent biocompatibility

We further investigated the *in vitro* and *in vivo* biocompatibility of ZH-1c-ENVs@Dex (**Additional Figure 3A**). In BV2 cells, co-incubation with ZH-1c-ENVs@Dex did not result in significant cytotoxicity. Cell viability remained above 90% in both treatment and control groups, as determined by CCK-8 assay (**Additional Figure 3B**). Consistently, Live/Dead fluorescence staining revealed predominantly green fluorescence with minimal red signal across all groups, indicating low levels of cell death (**Additional Figure 3C**).

*In vivo* safety was further assessed in healthy mice following tail vein injection of ZH-1c-ENVs@Dex. Body weight remained stable throughout the observation period, with no significant differences among groups (**Additional Figure 3D**). Complete blood count analysis revealed that the white blood cell count, red blood cell count, and platelet count remained within normal ranges in mice treated with ZH-1c-ENVs@Dex (**Additional Table 1**). Biochemical analysis of blood showed no significant differences in liver and kidney function indicators, including alanine aminotransferase, aspartate aminotransferase, blood urea nitrogen, and creatinine, between groups, suggesting no adverse effects on liver or kidney function (**Additional Figure 3E** and **F**). Additionally, H&E staining of major organs (liver, kidney, spleen, heart, and lungs) showed normal tissue structures with no pathological changes (**Additional Figure 3G**). These findings demonstrate that ZH-1c-ENVs@Dex exhibits excellent biocompatibility both *in vitro* and *in vivo*.

**Additional Table 1:** Complete blood count

Group	7 d			14 d			21 d		

	White blood cells	Red blood cells		White blood cells	Red blood cells		White blood cells	Red blood cells	
	(×10^3^/μL)	(×10^6^/μL)	Platelet (×10^3^/μL)	(×10^3^/μL)	(×10^6^/μL)	Platelet (×10^3^/μL)	(×10^3^/μL)	(×10^6^/μL)	Platelet (×10^3^/μL)
Control	7.2±0.6	8.1±0.5	310±25	7.3±0.5	8.1±0.3	313±22	7.0±0.5	7.8±0.3	305±25
ZH-1c-Exos@Dex	7.0±0.5	8.3±0.4	305±22	6.8±0.5	8.2±0.4	318±25	7.5±0.5	8.5±0.4	315±18

### ZH-1c-ENVs@Dex alleviates Alzheimer’s disease pathology by enhancing Sirtuin 3 expression and suppressing microglial inflammation and lysosomal overactivation

**Figure 4:**
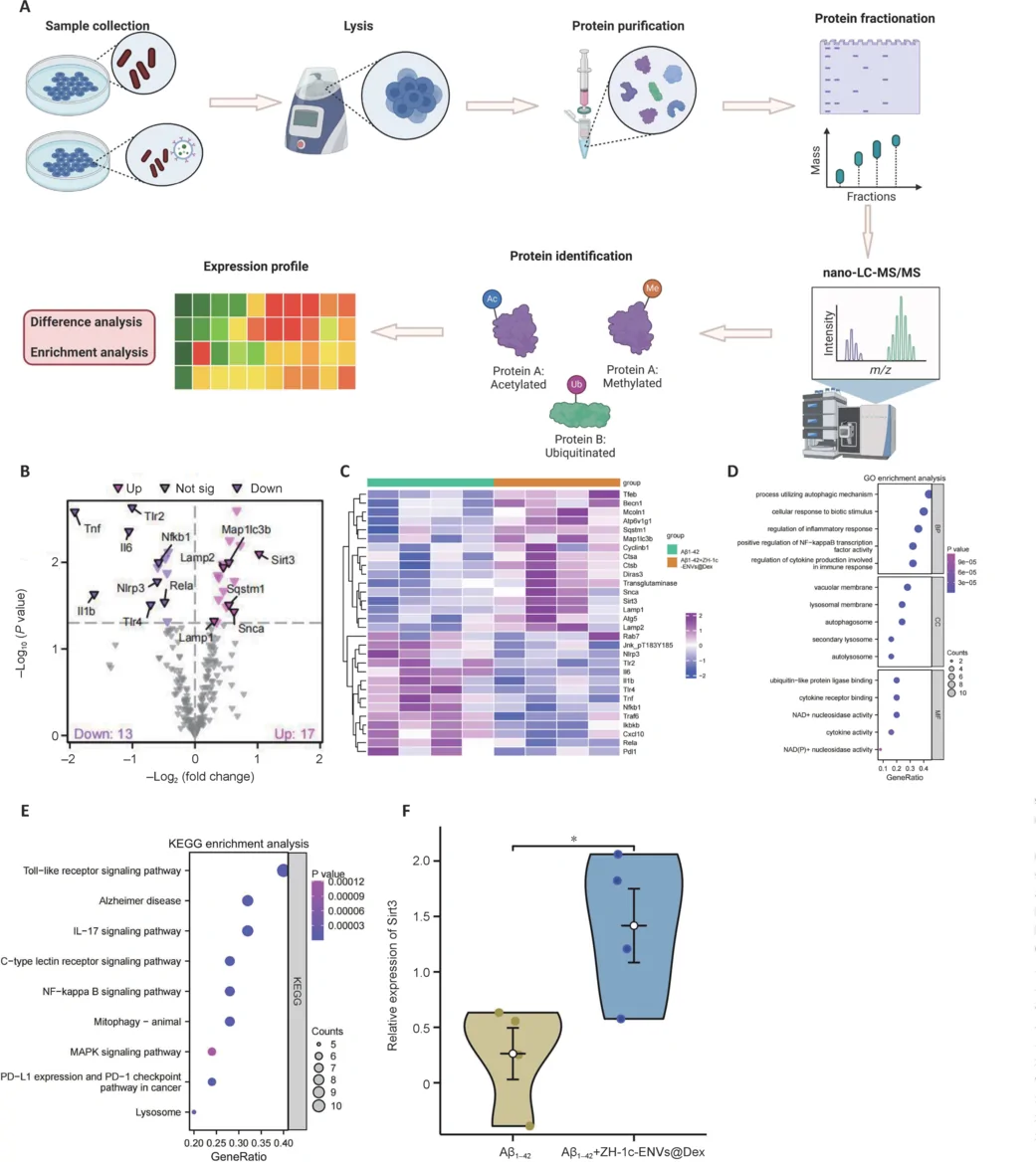
Bioinformatics analysis reveals the mechanism of ZH-1c-ENVs@Dex treatment in AD. (A) Workflow of protein sequencing. (B, C) Volcano plot (B) and heatmap (C) of DEPs in the AD and treatment groups (volcano plot: gray dots represent non-DEPs, red dots indicate upregulated proteins and purple dots indicate downregulated proteins, *n* = 4 independent experiments). (D) GO functional analysis of DEPs at the BP and CC levels. (E) KEGG pathway enrichment analysis of DEPs. (F) Expression levels of Sirt3 in different groups based on protein sequencing data. *P < 0.05, indicating statistical significance between groups. AD: Alzheimer’s disease; BP: biological process; CC: cellular component; DEPs: differentially expressed proteins; Dex: dexmedetomidine; ENVs: extracellular nanovesicles; GO: Gene Ontology; KEGG: Kyoto Encyclopedia of Genes and Genomes; Sirt3: Sirtuin 3.

To explore the underlying mechanisms, an *in vitro* AD model was established by treating microglial cells with Aβ_1–42_, followed by incubation with ZH-1c-ENVs@Dex for 24 hours. Proteomic sequencing was conducted on Aβ-treated cells and cells subsequently exposed to ZH-1c-ENVs@Dex (**Figure 4A**). Differential expression analysis identified 30 DEPs, comprising 17 upregulated and 13 downregulated proteins. The volcano plot (**Figure 4B**) revealed significant downregulation of inflammation-associated molecules, including Tlr2, Il6, Tnf, Nfkb1, Rela, and Nlrp3 (Li et al., 2023), indicating that Aβ-induced inflammatory pathways were activated but attenuated upon ZH-1c-ENVs@Dex treatment. Meanwhile, proteins involved in mitochondrial function and autophagy, such as Sirt3, Map1lc3b, and Lamp2, were differentially expressed, suggesting modulation of energy metabolism and lysosomal activity (Zhang et al., 2022; He et al., 2024). The heatmap (**Figure 4C**) showed clear clustering of these DEPs among groups, supporting the potential role of ZH-1c-ENVs@Dex in alleviating Aβ-induced cellular dysfunction. Functional enrichment analysis of the 30 DEPs was conducted using GO, which identified key BP, MF, and CC (**Figure 4D**). Enriched BP terms included “utilization of autophagic mechanisms,” “cellular response to biotic stimuli,” “modulation of inflammatory pathways,” and “upregulation of NF-κB transcriptional activity.” Enriched MF terms involved “ubiquitin-like protein ligase binding,” “cytokine receptor binding,” and “NAD^+^ nucleosidase activity.” The CC terms included “autophagosome,” “autolysosome,” and “secondary lysosome.” KEGG pathway analysis (**Figure 4E**) revealed significant involvement in Toll-like receptor signaling, NF-κB activation, lysosomal processes, MAPK cascades, and AD-related pathways, indicating that ZH-1c-ENVs@Dex may act through multiple inflammation- and lysosome-associated mechanisms.

Sirt3, a NAD^+^-dependent mitochondrial deacetylase, has been widely reported to regulate oxidative stress, mitochondrial homeostasis, and autophagy (Zhang et al., 2025). Prior studies have shown that Dex can enhance Sirt3 expression and reduce neuroinflammation (Zhang et al., 2021; He et al., 2023; Lu et al., 2024). Proteomic analysis revealed that Sirt3 was the most significantly upregulated protein following ZH-1c-ENVs@Dex treatment (**Figure 4F**). Additionally, members of the Sirtuin family are known to promote neuroprotection by modulating microglial activation and inhibiting Aβ aggregation, thereby preventing AD progression (Eshraghi et al., 2021; Watroba and Szukiewicz, 2022; Zhou et al., 2024). These findings suggest that ZH-1c-ENVs@Dex may exert therapeutic effects in AD by promoting Sirt3 expression, subsequently inhibiting microglial inflammatory responses and abnormal lysosomal activation, and ultimately ameliorating Aβ-induced neurotoxicity.

### ZH-1c-ENVs@Dex suppresses microglial inflammation and enhances lysosomal activity for clearing amyloid-beta aggregates through the activation of Sirtuin 3

To further validate these effects, we investigated the impact of ZH-1c-ENVs@Dex on microglial activation and lysosomal clearance of Aβ aggregates *in vitro*. Aβ_42_ peptides were used to prepare oligomers and fibrils, while Aβ40 peptides served as monomers (**Additional Figure 4A**). Cells treated with Aβ oligomers and fibrils showed that fibrils were less effective in inducing microglial activation and lysosomal dysfunction than oligomers (**Additional Figure 4B**).

**Figure 5:**
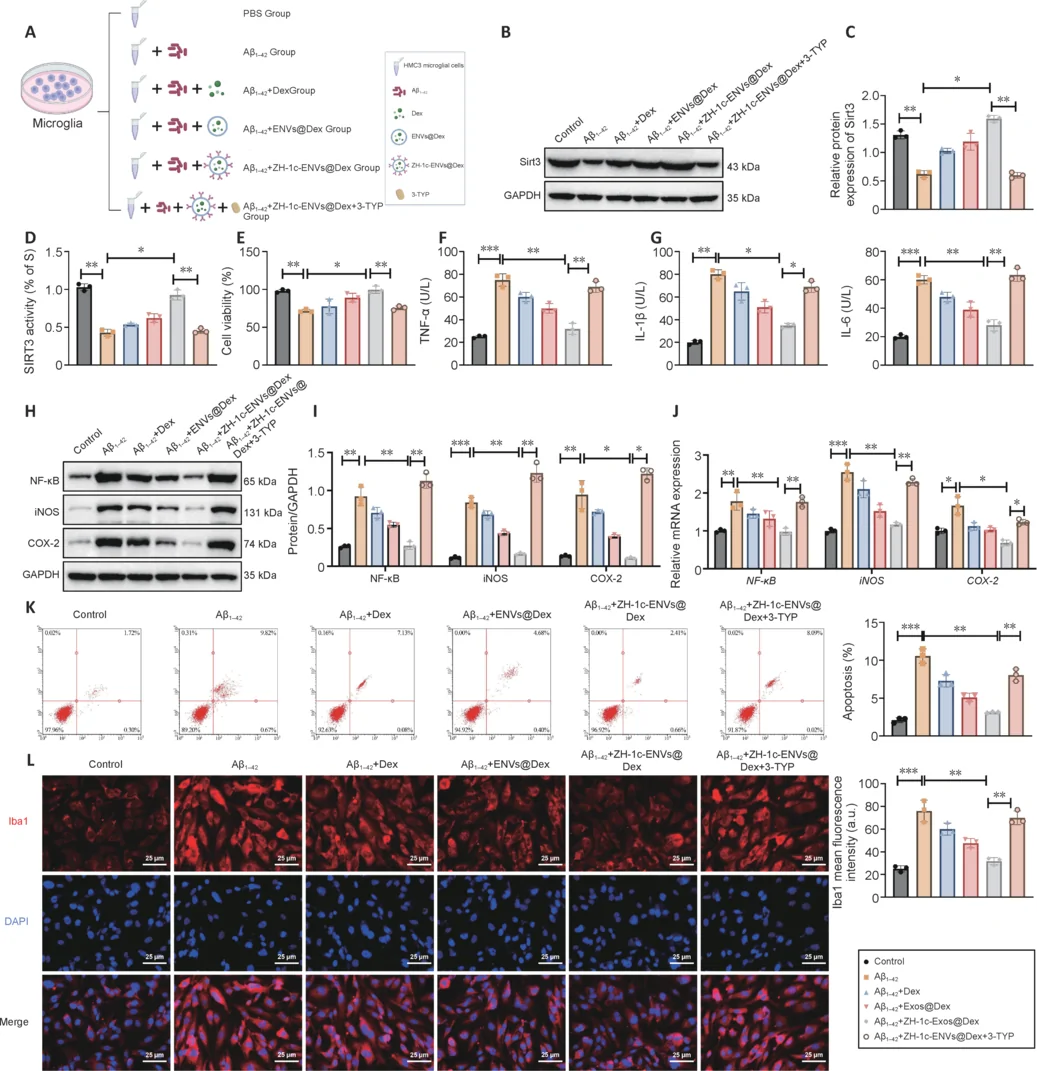
Effects of ZH-1c-ENVs@Dex on microglial activation. (A) Workflow for constructing an *in vitro* Aβ_1–42_-treated microglial activation model. (B, C) WB analysis of Sirt3 expression levels in different groups. (D) Sirt3 activity assessed using fluorescence detection. (E) Microglial viability evaluated by Cell Counting Kit-8 assay. (F, G) Enzyme-linked immunosorbent assay for pro-inflammatory cytokines (TNF-α, IL-1β, and IL-6) expression levels. (H, I) WB analysis of NF-κB, iNOS, and COX-2 expression levels. (J) Quantitative reverse transcription-polymerase chain reaction analysis of *NF-κB*, *iNOS*, and *COX-2* mRNA levels. (K) Apoptosis rates of microglial cells assessed by flow cytometry. (L) Immunofluorescence staining for the microglial activation marker Iba1. Scale bars: 25 μm. Data in C, D–G, I–K are presented as mean ± SD. Cellular experiments were performed in triplicate. *P < 0.05, **P < 0.01, ***P < 0.001. 3-TYP: Sirt3 inhibitor; Aβ: amyloid-beta; DAPI: 4′,6-diamidino-2-phenylindole; Dex: dexmedetomidine; ENVs: extracellular nanovesicles; GAPDH: glyceraldehyde-3-phosphate dehydrogenase; Iba1: ionized calcium-binding adapter molecule 1; IL: interleukin; iNOS: inducible nitric oxide synthase; NF-κB: nuclear factor kappa B; Sirt3: Sirtuin 3; TNF-α: tumor necrosis factor-α; WB: western blot.

Therefore, subsequent experiments were conducted using Aβ oligomers. The experimental groups included: Control, Aβ_1–42_, Aβ_1–42_ + Dex, Aβ_1–42_ + ENVs@Dex, Aβ_1–42_ + ZH-1c-ENVs@Dex, and Aβ_1–42_ + ZH-1c-ENVs@Dex + 3-TYP (**Figure 5A**). Western blotting (WB) and fluorescence assays revealed that both the expression and activity of Sirt3 were significantly decreased in the Aβ_1–42_ group relative to the control group. In contrast, treatment with Dex, ENVs@Dex, or ZH-1c-ENVs@Dex markedly restored Sirt3 expression and activity, with ZH-1c-ENVs@Dex producing the most pronounced upregulation. Notably, this upregulation was reversed upon co-treatment with the Sirt3-specific inhibitor 3-TYP (**Figure 5B–D**). Cell viability assessed via the CCK-8 assay revealed a marked decline in the Aβ_1–42_-treated group compared to the control group. Conversely, the Aβ_1–42_ + Dex, Aβ_1–42_ + ENVs@Dex, and Aβ_1–42_ + ZH-1c-ENVs@Dex groups exhibited significantly enhanced microglial viability, with the Aβ_1__–42_ + ZH-1c-ENVs@Dex group displaying the highest viability. This increase in cell viability was reversed by the addition of 3-TYP (**Figure 5E**).

ELISA results demonstrated that exposure to Aβ_1–42_ led to a significant elevation in the release of pro-inflammatory cytokines, including TNF-α, IL-1β, and IL-6, when compared to the control group. Treatment with Dex, ENVs@Dex, or ZH-1c-ENVs@Dex effectively reduced cytokine levels, with ZH-1c-ENVs@Dex producing the most substantial suppression. Notably, the anti-inflammatory effect of ZH-1c-ENVs@Dex was significantly reversed by co-administration of the Sirt3 inhibitor 3-TYP (**Figure 5F** and **G**). Further analysis using qRT-PCR and WB revealed a marked upregulation of inflammatory signaling mediators (NF-κB, iNOS, and COX-2) in the Aβ_1–42_ group relative to the Control. These pro-inflammatory markers were significantly downregulated following treatment with Dex, ENVs@Dex, or ZH-1c-ENVs@Dex, with the most prominent decrease observed in the ZH-1c-ENVs@Dex group. Again, these effects were abolished by 3-TYP (**Figure 5H–J**). FCM analysis revealed that Aβ_1–42_ induced a significant increase in microglial apoptosis. In contrast, Dex, ENVs@Dex, and ZH-1c-ENVs@Dex significantly reduced apoptosis rates, with ZH-1c-ENVs@Dex exerting the strongest protective effect. This anti-apoptotic effect was significantly mitigated by 3-TYP (**Figure 5K**). Immunofluorescence staining of the microglial activation marker Iba1 showed elevated expression in the Aβ_1–42_ group. However, the Aβ_1–42_ + Dex, Aβ_1–42_ + ENVs@Dex, and Aβ_1–42_ + ZH-1c-ENVs@Dex groups demonstrated significantly reduced Iba1 expression, with the Aβ_1–42_ + ZH-1c-ENVs@Dex group exhibiting the most pronounced inhibition. This effect was also reversed by 3-TYP (**Figure 5L**).

To further elucidate the regulatory role of ZH-1c-ENVs@Dex on microglial biology, we investigated its effects on lysosomal function. Microglial cells exposed to Aβ_1–42_ displayed pronounced lysosomal impairment, as evidenced by reduced expression of the lysosomal membrane protein LAMP1 and decreased levels of key lysosomal enzymes, including CatB and V-ATPase. Treatment with Dex, ENVs@Dex, or ZH-1c-ENVs@Dex partially restored the expression of these markers, indicating amelioration of lysosomal dysfunction. Among them, ZH-1c-ENVs@Dex treatment led to the highest levels of LAMP1 expression and lysosomal enzyme activity, suggesting superior restoration of lysosomal function. These restorative effects were significantly attenuated by co-treatment with the Sirt3 inhibitor 3-TYP (**Additional Figure 5A**). Additionally, we used the green LysoSensor probe to assess lysosomal abundance in cells. Immunofluorescence results revealed a marked reduction in lysosomal number in microglial cells stimulated with Aβ_1–42_ relative to the control group. Treatment with Dex, ENVs@Dex, or ZH-1c-ENVs@Dex significantly increased lysosomal numbers, with ZH-1c-ENVs@Dex producing the most pronounced effect. Again, this enhancement was reversed by 3-TYP (**Additional Figure 5B**). In summary, in AD mice, overall LAMP1 protein expression was decreased; however, immunofluorescence showed an increase in LAMP1 puncta within activated microglia, suggesting aggregation of residual lysosomes. Further analysis revealed spatial colocalization between LAMP1-positive puncta and Iba1-positive cells (**Additional Figure 5B**), indicating that microglial activation may be accompanied by structural changes in lysosomes.

These findings collectively demonstrate that ZH-1c-ENVs@Dex effectively reverses Aβ_1–42_-induced lysosomal dysfunction in microglial cells by stimulating Sirt3 activity, leading to increased lysosomal numbers and enhanced enzyme secretion.

### ZH-1c-ENVs@Dex activates Sirtuin 3 to enhance lysosome-mediated amyloid-beta clearance in microglial cells *in vitro* and *in vivo*

**Figure 6:**
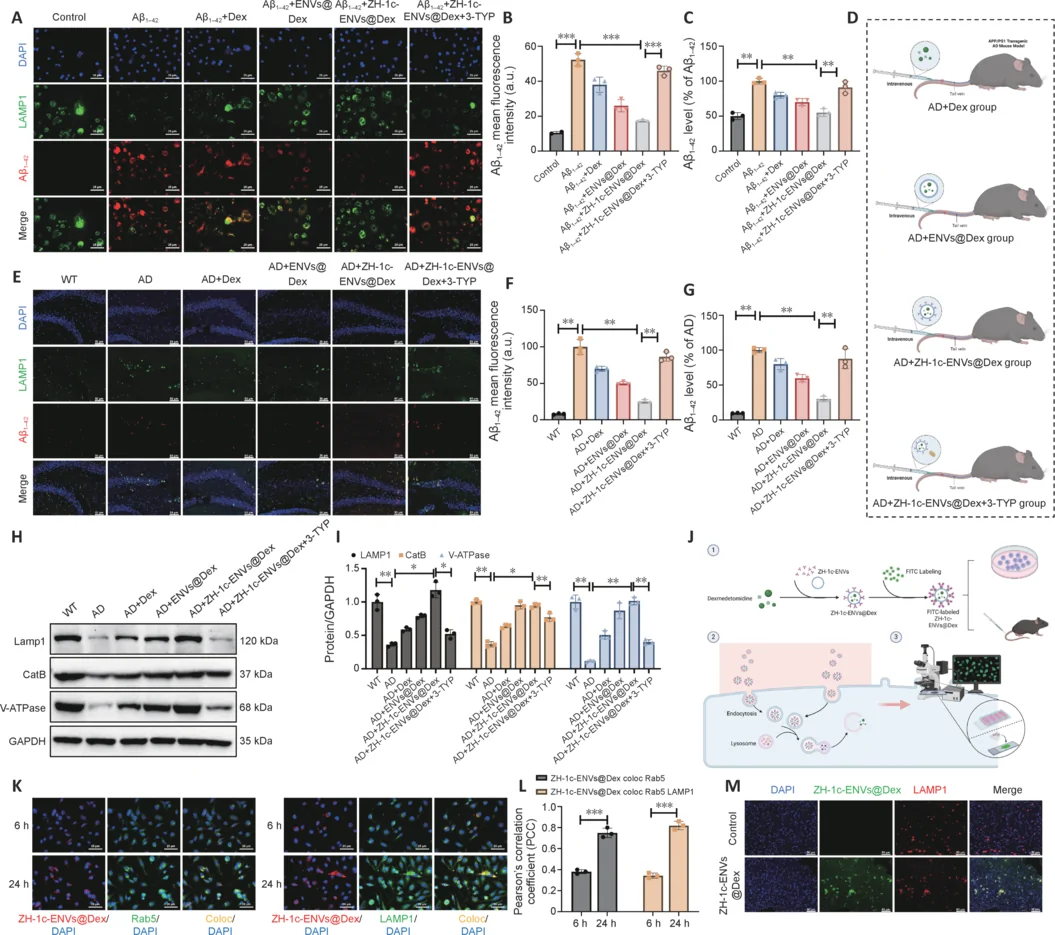
Effects of ZH-1c-ENVs@Dex on Aβ_1–42_ clearance *in vitro* and *in vivo*. (A) Fluorescence images showing colocalization of lysosomes and Aβ_1–42_ in microglial cells after Aβ_1–42_ treatment, scale bars: 25 μm. (B) Statistical analysis of Aβ_1–42_ expression levels. (C) ELISA results quantifying Aβ_1–42_ content in microglial cells for each group. (D) Schematic diagram of AD mouse group allocation. (E) Immunofluorescence images of Aβ_1–42_ plaques colocalized with lysosomes in the hippocampal region of AD mice, scale bars: 50 μm. (F) Fluorescence quantification of Aβ_1–42_ plaques in the hippocampus after injection of ENVs, Dex, ENVs@Dex, or ZH-1c-ENVs@Dex. (G) ELISA quantification of hippocampal Aβ_1–42_ expression levels following different treatments. (H, I) Western blot analysis of Lamp1, CatB, and V-ATPase expression in the hippocampus of AD mice. (J) Schematic illustration of immunofluorescence images of tissues from mice and cells treated with FITC-labeled ZH-1c-ENVs@Dex. (K) Immunofluorescence images showing colocalization of Rab5 and Lamp1 in cells treated with FITC-labeled ZH-1c-ENVs@Dex, scale bars: 25 μm. (L) Statistical analysis of colocalization between ZH-1c-ENVs@Dex and lysosomal markers Rab5 and Lamp1. (M) Immunofluorescence images showing colocalization of FITC-labeled ZH-1c-ENVs@Dex with Lamp1 in tissues, scale bars: 50 μm. Data in B, C, F, G, I, L are presented as mean ±SD. Experiments were repeated three times. *P < 0.05, **P < 0.01. 3-TYP: Sirtuin 3 inhibitor; AD: Alzheimer’s disease; Aβ: amyloid-beta; CatB: cathepsin B; Coloc: colocalization; DAPI: 4′,6-diamidino-2-phenylindole; Dex: dexmedetomidine; ELISA: enzyme-linked immunosorbent assay; ENVs: extracellular nanovesicles; GAPDH: glyceraldehyde-3-phosphate dehydrogenase; WT: wild-type.

To investigate whether the restoration of lysosomal activity by ZH-1c-ENVs@Dex contributes to Aβ clearance, we evaluated lysosomal activity in microglial cells pretreated with Aβ_1–42_ and subsequently exposed to Dex, ENVs@Dex, or ZH-1c-ENVs@Dex. Immunofluorescence analysis revealed substantial colocalization between lysosomes and Aβ_1–42_ aggregates, indicating that Aβ_1–42_ was efficiently internalized by microglial cells and trafficked to the lysosomal compartment for degradation. In contrast, Aβ_1–42_ treatment alone markedly reduced Lamp1 expression compared to controls and led to the accumulation of Aβ_1–42_, suggesting that Aβ_1–__42_ induces lysosomal dysfunction in microglial cells, thereby impairing Aβ clearance. Following treatment with Dex, ENVs@Dex, or ZH-1c-ENVs@Dex, lysosomal activity was significantly restored, and Aβ_1–42_ fluorescence intensity was substantially reduced. Notably, treatment with 3-TYP reversed the effects of ZH-1c-ENVs@Dex on lysosomal dysfunction in microglial cells (**Figure 6A** and **B**). Furthermore, ELISA results confirmed that ZH-1c-ENVs@Dex exhibited the most pronounced effect in enhancing Aβ_1–42_ clearance, an effect that was reversed by 3-TYP treatment (**Figure 6C**). To further assess the intracellular dynamics of Aβ clearance, time-lapse imaging was performed using FITC-labeled Aβ_1–42_ in BV2 cells treated with ZH-1c-ENVs@Dex. The imaging data showed progressive uptake of FITC-Aβ_1–__42_ followed by a gradual decrease in intracellular fluorescence intensity, indicating effective degradation and clearance over time (**Additional Figure 6A**).

We further validated the effects of ZH-1c-ENVs@Dex *in vivo*. Mice were divided into six groups: WT, AD, AD + Dex, AD + ENVs@Dex, AD + ZH-1c-ENVs@Dex, and AD + ZH-1c-ENVs@Dex + 3-TYP (**Figure 6D**). The study focused on the clearance of Aβ_1–42_ in the hippocampus of AD model mice mediated by ZH-1c-ENVs@Dex. WB and fluorescence analyses demonstrated a decrease in Sirt3 expression and activity in the hippocampi of AD mice compared to WT controls. Treatment with Dex, ENVs@Dex, or ZH-1c-ENVs@Dex markedly restored Sirt3 expression and enzymatic activity, with the most pronounced effect observed in the ZH-1c-ENVs@Dex group. These improvements were effectively abrogated by co-administration of the Sirt3 inhibitor 3-TYP (**Additional Figure 6B–D**). TEM further revealed that ZH-1c-ENVs@Dex substantially preserved the ultrastructural integrity of microglial mitochondria, which was otherwise severely compromised in the AD group (**Additional Figure 6E**), reinforcing the central role of Sirt3 in maintaining mitochondrial homeostasis. In addition, immunofluorescence staining and ELISA confirmed extensive deposition of Aβ_1–42_ plaques in the hippocampus of AD mice, accompanied by colocalization with lysosomes. Following systemic administration of Dex, ENVs@Dex, or ZH-1c-ENVs@Dex, the burden of Aβ_1–42_ plaques was significantly reduced, with the ZH-1c-ENVs@Dex group exhibiting the lowest plaque density and size. Additionally, LAMP1 expression was markedly elevated in the ZH-1c-ENVs@Dex group (**Figure 6E–G**). We also assessed the lysosomal activity in hippocampal microglial cells of AD model mice treated with ZH-1c-ENVs@Dex. In the AD model, microglial cells exhibited lysosomal dysfunction, characterized by reduced levels of the lysosomal marker protein Lamp1 and downregulated expression of CatB and V-ATPase hydrolases. Treatment with Dex, ENVs@Dex, or ZH-1c-ENVs@Dex partially restored the expression of these key lysosomal components. Among the treatment groups, ZH-1c-ENVs@Dex achieved the most robust enhancement in both LAMP1 expression and hydrolase secretion (**Figure 6H** and **I**). The addition of 3-TYP reversed the effects of ZH-1c-ENVs@Dex on AD model mice, further confirming the role of Sirt3.

To examine the lysosomal targeting potential of ZH-1c-ENVs@Dex, we labeled the nanovesicles with FITC and administered them to both cells and SD rats. Immunofluorescence staining for lysosomal markers Rab5 and LAMP1 revealed a notable increase in co-localization between FITC-labeled ZH-1c-ENVs@Dex and lysosomes, as shown in **Figure 6J**. Quantitative analysis further confirmed significant colocalization in both animal and cellular models (**Figure 6K–M**), indicating effective lysosomal trafficking and suggesting a direct interaction.

These findings indicate that ZH-1c-ENVs@Dex can activate Sirt3, undergo endocytosis into microglial cells, and be transported to lysosomes. This process suppresses microglial inflammation, enhances lysosomal activity within microglial cells, and accelerates the clearance of Aβ_1–42_ both *in vivo* and *in vitro*.

### ZH-1c-ENVs@Dex activates Sirtuin 3 to improve cognitive function in Alzheimer’s disease mice

**Figure 7:**
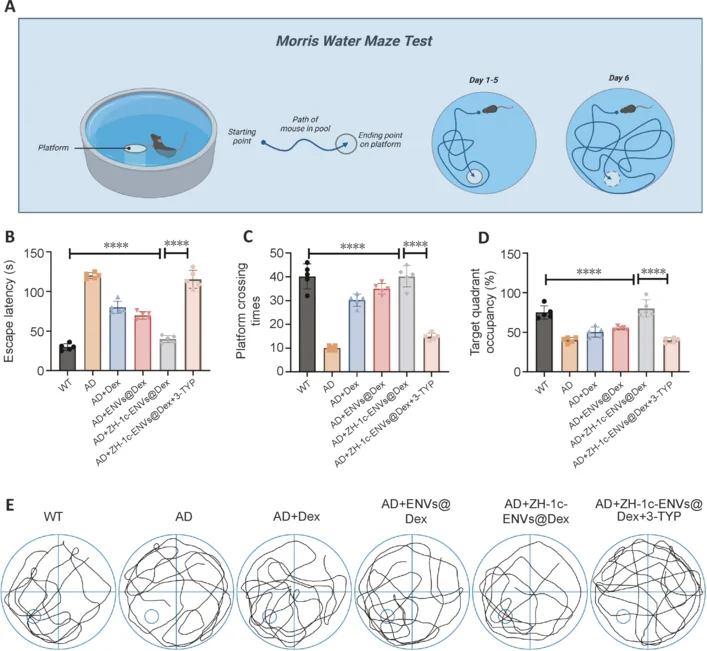
Effects of ZH-1c-ENVs@Dex on cognitive function assessed using the MWM in AD mice. (A) Schematic diagram of the experimental procedure in AD mice. (B) Escape latency of mice in different treatment groups during the MWM test. (C) Number of platform crossings by mice in different treatment groups on day 6. (D) Percentage of time spent in the target quadrant by mice in different treatment groups on day 6. (E) Representative swimming paths of mice in different treatment groups on day 6. Data in B–D are presented as mean ± SD. *P < 0.05, **P < 0.01, ***P < 0.001; *n* = 5 animals per group. AD: Alzheimer’s disease; Aβ: amyloid-beta; Dex: dexmedetomidine; ENVs: extracellular nanovesicles; MWM: Morris water maze; WT: wide type.

The therapeutic efficacy of ZH-1c-ENVs@Dex in restoring cognitive function was evaluated in AD mice using the MWM test (**Figure 7A**). During the 5-day training period, AD mice treated with Dex, ENVs@Dex, or ZH-1c-ENVs@Dex exhibited shorter escape latencies compared to the untreated AD group. Notably, the ZH-1c-ENVs@Dex treatment group showed significantly shorter escape latencies than the Dex and ENVs@Dex groups, nearly reaching the level of WT controls. However, this improvement was reversed upon administration of 3-TYP (**Figure 7B**). To assess the durability of the cognitive improvements, MWM tests were repeated six weeks post-treatment. Mice in the ZH-1c-ENVs@Dex group continued to show enhanced performance, indicating sustained neuroprotective effects (**Additional Figure 7A–D**).

On the sixth day of testing, following removal of the platform, mice in the ZH-1c-ENVs@Dex group displayed a significantly higher number of platform crossings and spent more time in the target quadrant compared to those in the Dex and ENVs@Dex groups (**Figure 7C** and **D**). Representative swimming traces (**Figure 7E**) further confirmed that ZH-1c-ENVs@Dex-treated mice focused their search near the previous platform location. These improvements were abrogated by 3-TYP, supporting the critical role of Sirt3 activation. Together, these findings indicate that ZH-1c-ENVs@Dex significantly alleviates cognitive impairment in AD mice by enhancing spatial learning and memory, effects that are dependent on Sirt3 activation.

**Figure 8:**
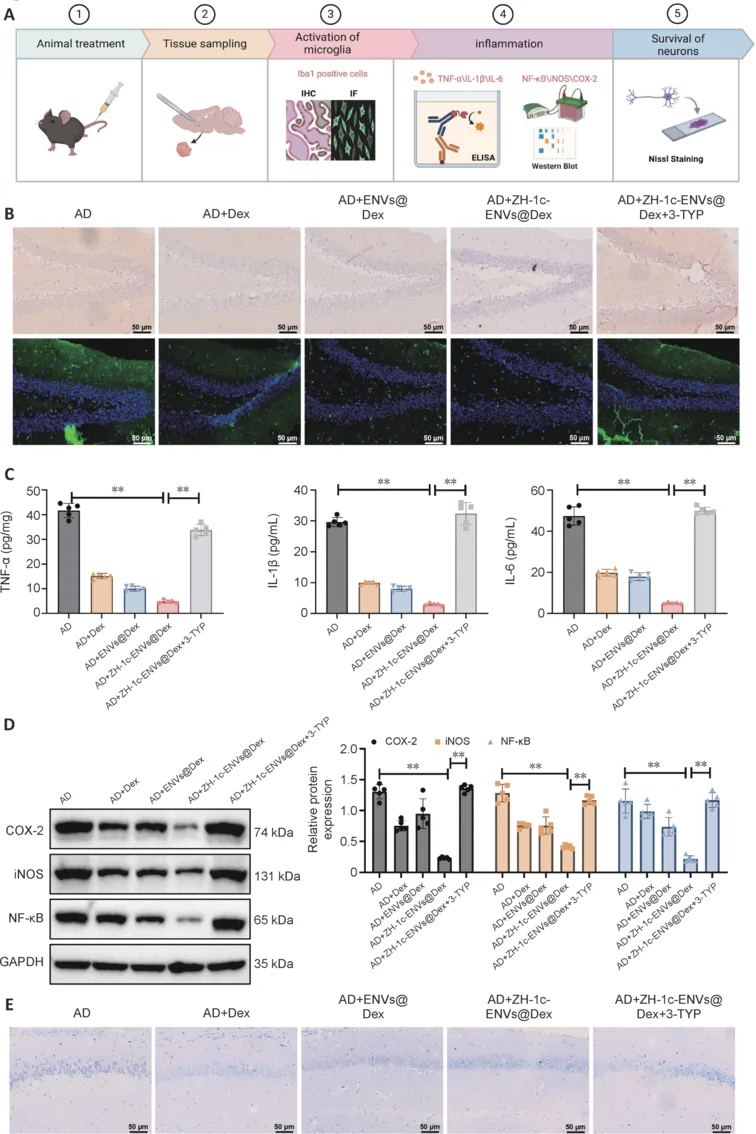
Effects of ZH-1c-ENVs@Dex on hippocampal inflammation and neuronal survival in the brains of AD mice. (A) Schematic workflow of experiments investigating hippocampal inflammation and neuronal survival in the brains of AD mice. (B) Immunohistochemical and immunofluorescence staining showing the number of Iba1-positive cells in the hippocampus across different treatment groups (scale bars: 50 μm). (C) Enzyme-linked immunosorbent assay results quantifying inflammatory cytokines (TNF-α, IL-1β, and IL-6) in the hippocampus across different treatment groups. (D) Western blot analysis of inflammatory signaling pathway-related proteins (NF-κB, iNOS, and COX-2) in the hippocampus across different treatment groups. (E) Nissl staining to assess neuronal survival in the hippocampus across different treatment groups (scale bars: 50 μm). Data in C and D are presented as mean ± SD. *P < 0.05, **P < 0.01; *n* = 5 animals per group. 3-TYP: Sirtuin 3 inhibitor; AD: Alzheimer’s disease; COX-2: cyclooxygenase-2; Dex: dexmedetomidine; ENVs: extracellular nanovesicles; GAPDH: glyceraldehyde-3-phosphate dehydrogenase; IL: interleukin; iNOS: inducible nitric oxide synthase; NF-κB: nuclear factor kappa B; TNF-α: tumor necrosis factor-α.

The activation state of microglial cells and the expression of neuroinflammatory markers in the hippocampal region of brain tissue were assessed using immunohistochemistry and immunofluorescence staining (**Figure 8A**). A significant reduction in Iba1-positive microglia was observed in the Dex, ENVs@Dex, and ZH-1c-ENVs@Dex treatment groups compared to the untreated AD group, with the ZH-1c-ENVs@Dex group showing the most pronounced suppression. This effect was reversed upon administration of the Sirt3 inhibitor 3-TYP (**Figure 8B**). ELISA analysis revealed that the levels of inflammatory cytokines TNF-α, IL-1β, and IL-6 were lower in the Dex, ENVs@Dex, and ZH-1c-ENVs@Dex groups, with the greatest reduction observed in the ZH-1c-ENVs@Dex group. Similarly, 3-TYP reversed these reductions in inflammatory cytokine levels (**Figure 8C**). WB analysis of NF-κB, iNOS, and COX-2 expression in the hippocampal region corroborated the ELISA findings, showing a consistent trend (**Figure 8D**). Given that Aβ_1–42_ accumulation induces pronounced neurotoxicity and neuronal apoptosis, we next investigated whether ZH-1c-ENVs@Dex could attenuate Aβ_1–42_-induced neuronal injury. Nissl staining revealed that treatment with ZH-1c-ENVs@Dex significantly increased the number of surviving neurons and preserved hippocampal neuronal architecture compared to other treatment groups. Again, 3-TYP reversed these neuroprotective effects (**Figure 8E**). These findings confirm that ZH-1c-ENVs@Dex induces the highest neuronal survival rate and exhibits the strongest anti-apoptotic effects by activating Sirt3. These findings suggest that ZH-1c-ENVs@Dex effectively enhances lysosomal activity in microglia and improves neuronal survival.

## Discussion

AD is a complex neurodegenerative disorder in which neuroinflammation plays a critical role in its pathological progression (Chen and Yu, 2023; Twarowski and Herbet, 2023; Adamu et al., 2024). This study aims to develop an aptamer-modified nanovesicle delivery system (ZH-1c-ENVs@Dex) to target microglial cells and modulate their function, thereby alleviating AD pathology. In contrast to previous strategies, this platform employs lipid conjugation to achieve robust aptamer stability, thereby enabling ZH-1c-ENVs to efficiently cross the BBB and undergo selective endocytosis by microglia. This design significantly enhances the targeting specificity of the drug delivery system, providing a novel paradigm for the treatment of CNS diseases while addressing the limitations of traditional delivery systems in terms of specificity and stability.

The core innovation of ZH-1c-ENVs@Dex lies in its regulation of microglial function through the activation of the Sirt3 signaling pathway (Yu et al., 2020). While Dex has been widely recognized as a potent anti-inflammatory agent, our findings further demonstrate that Dex enhances lysosomal activity and suppresses inflammatory responses in microglial cells, with Sirt3 playing a central role in mediating these effects. Notably, this study is the first to implicate the Sirt3 signaling axis in the anti-inflammatory action of Dex, thus expanding its therapeutic relevance in neurodegenerative diseases. Although no direct interaction between Dex and Sirt3 has been reported to date, it is likely that Dex modulates Sirt3 indirectly via upstream metabolic regulators, particularly the AMPK–PGC-1α pathway (Yan et al., 2024). For instance, Dex has been shown to upregulate PGC-1α expression, thereby promoting Sirt3 transcription and protein levels. In a renal ischemia-reperfusion model, Dex treatment markedly increased both PGC-1α and Sirt3 expression (Koyuncu et al., 2025). Moreover, AMPK activation can upregulate PGC-1α, and PGC-1α overexpression enhances Sirt3 mRNA levels (Gu et al., 2023; Li et al., 2025). Sirt3 is well-established as a key regulator of microglial phenotypic switching, and its downstream effectors, including FoxO3a and PGC-1α, are involved in cellular energy metabolism, reactive oxygen species clearance, and the modulation of neuroinflammation in central nervous system disorders (Wu et al., 2023; Li et al., 2024). In the present study, Dex treatment increased Sirt3 expression in both AD model mice and BV2 microglial cells, which was accompanied by suppression of the inflammatory phenotype and a reduction in Aβ burden. These results strongly support the functional relevance of the Sirt3 axis in the AD microenvironment. To our knowledge, this is the first report to propose an “Aptamer–Nanovesicle–Dex–Sirt3” axis as a therapeutic framework for AD, offering a novel conceptual model for microglial modulation. Moving forward, we plan to employ co-immunoprecipitation, Sirt3 overexpression/knockdown, and downstream target intervention to further elucidate the Dex-Sirt3 axis and identify its key regulatory nodes. The elucidation of this complex mechanism not only validates the therapeutic value of Dex but also enhances our understanding of the molecular basis of microglial dysfunction.

The mutual validation of *in vitro* and *in vivo* experiments provides robust data to support this study. In the BV2 microglial cell model, ZH-1c-ENVs@Dex was effectively internalized by the cells, significantly suppressing the expression of inflammation-related genes and enhancing lysosomal activity. *In vivo* studies further demonstrated that ZH-1c-ENVs@Dex markedly improved cognitive function in an AD mouse model, as verified by the MWM test. These findings suggest that ZH-1c-ENVs@Dex exhibits pronounced anti-inflammatory and neuroprotective effects on multiple levels, offering greater comprehensiveness and efficacy compared to traditional therapeutic strategies that rely on single mechanisms.

The primary advantages of ZH-1c-ENVs@Dex over conventional therapies lie in its targeted delivery and multifunctional regulatory capacity. Current clinical interventions for AD mainly alleviate symptoms without addressing the underlying pathophysiological mechanisms. By specifically targeting microglial cells, ZH-1c-ENVs@Dex minimizes off-target effects and enables coordinated regulation through the Sirt3 signaling axis. This nanotechnology-based precision strategy provides a promising framework for the development of next-generation therapeutics for CNS disorder.

Additionally, proteomic analysis offered crucial molecular insights underpinning the observed therapeutic effects. High-throughput profiling of protein expression in microglial cells treated with ZH-1c-ENVs@Dex identified Sirt3 as a key regulatory node and revealed potential downstream effectors. The integration of proteomic data with functional assays provides a more holistic understanding of microglial dysfunction in AD, surpassing traditional approaches that rely primarily on gene-level or single-marker validation. This comprehensive mechanistic perspective lays a solid foundation for future mechanistic and translational studies.

This research introduces an innovative nanodrug delivery system that enhances the targeting ability of ENVs through aptamer modification and leverages the anti-inflammatory properties of Dex to effectively inhibit microglial activation. The system exhibited outstanding therapeutic effects in an AD model, suggesting its potential as a new strategy for treating AD and other neuroinflammation-related disorders. Furthermore, as natural nanocarriers, ENVs possess excellent biocompatibility and low immunogenicity, further supporting their potential for clinical application.

Despite the promising outcomes, several limitations should be acknowledged. The experimental framework predominantly relied on AD mouse models and BV2 murine microglial cells, without verification in human-based systems. The lack of validation using human induced pluripotent stem cell-derived microglia, brain tissues from AD patients, or non-human primate models reduces the translational impact of the current findings. To strengthen clinical relevance, future research will incorporate induced pluripotent stem cell-derived human microglia and patient-derived samples to further assess the therapeutic potential of this strategy. Additionally, although aptamer-mediated BBB targeting exhibits remarkable potential, it faces certain limitations, such as receptor saturation that may reduce delivery efficiency under repeated or high-dose administration, and possible immunogenicity of some aptamers, which could affect safety and long-term stability. Compared with lipid nanoparticles, which offer good biocompatibility and drug-loading capacity but suffer from nonspecific BBB distribution, and exosomes, which are naturally biocompatible and immune-evasive yet limited in loading efficiency and engineering controllability, the aptamer-mediated system provides distinct advantages in targeting specificity and delivery stability. Nonetheless, further optimization in aptamer screening, modification strategies, and delivery efficiency is essential for broader application in CNS therapeutics. Although this study elucidates the pivotal mechanism by which Dex regulates microglial function through the Sirt3 signaling pathway, the potential crosstalk with other signaling pathways remains incompletely understood. Moreover, the preparation and purification of ENVs are relatively complex and costly, highlighting the need for further optimization and simplification. In addition, as the present work was primarily conducted in a mouse model, larger-scale animal studies and clinical trials are required to validate the safety and efficacy of this strategy. Future research could focus on enhancing this approach through multi-aptamer co-modification or integration of multi-omics data, as well as conducting comprehensive long-term pharmacological and toxicological assessments to strengthen its translational potential. Furthermore, improving the manufacturing process of ENVs to reduce production costs and verifying their therapeutic efficacy across diverse neuroinflammatory models will be crucial. Exploration of other anti-inflammatory agents or therapeutic molecules in combination with ENVs may also provide promising alternatives. Collectively, the strategy of aptamer-modified ENVs for drug delivery holds broad application prospects in the treatment of neuroinflammatory diseases.

**Figure 9:**
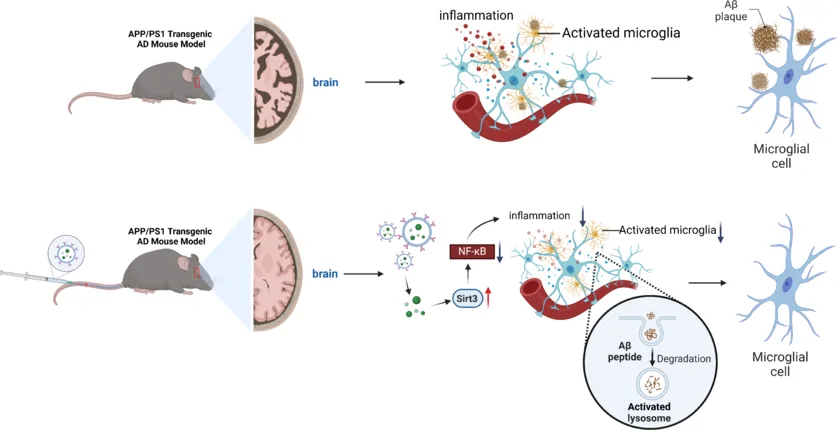
Schematic representation of the molecular mechanism by which ZH-1c-ENVs@Dex suppress microglial inflammation, enhance lysosomal activity for Aβ aggregation clearance, and mitigate neuroinflammatory damage in AD mice. AD: Alzheimer’s disease; Aβ: amyloid-beta; Dex: dexmedetomidine; ENVs: extracellular nanovesicles; NF-κB: nuclear factor kappa B; Sirt3: Sirtuin 3.

In conclusion, this study successfully developed and characterized aptamer-modified ENVs loaded with Dex, demonstrating their stability and drug release behaviors both *in vitro* and *in vivo*. The aptamer-modified ENVs exhibited excellent physical stability under various conditions and showed controlled drug release profiles under different pH environments (**Figure 9**). Experimental results indicated that these ENVs effectively suppressed microglial activation, significantly reduced inflammatory cytokine levels, and alleviated neuroinflammatory damage in AD mouse models. The ZH-1c-ENVs@Dex system holds promise as a novel nanomedicine strategy for the targeted regulation of AD-related neuroinflammation and cognitive impairment, demonstrating potential for future therapeutic translation.

## Additional files:

***Additional Figure 1:*** *Labeling efficiency of ZH-1c-PEG*_*2000*_*-chol on microglial cell membranes and cell amplification.*

Additional Figure 1 Labeling efficiency of ZH-1c-PEG_2000_-chol on microglial cell membranes and cell amplification.
(A) Microglial cells were incubated with FITC-labeled chol-PEG_2000_ in concentrations ranging from 0 to 5 nmol. After labeling, cells were cultured into spheroids, and the fluorescence intensity of the supernatant was measured. (B) Fluorescence intensity of FITC-labeled chol-PEG_2000_ before and after cell labeling. (C) Quantification of lipids incorporated into cell membranes across groups. (D) Schematic representation of isolated and amplified microglial cells cultured *in vitro* for 5 days. (E) CD11c-labeled analysis of the purity of isolated microglial cells. FITC: Fluorescein isothiocyanate; PEG: polyethylene glycol.

***Additional Figure 2:*** *Preparation and characterization of ZH-1c aptamer-modified ENVs.*

Additional Figure 2 Preparation and characterization of ZH-1c aptamer-modified ENVs.
(A) Western blot analysis of cytoplasmic and surface protein expression in ZH-1c-ENVs and ENVs. (B) Stability assessment of ZH-1c-ENVs and ENVs after 6 months of storage at -80°C using dynamic light scattering. (C) Verification of ZH-1c conjugation on ENVs via fluorescence dot blot assay. (D) Fluorescence microscopy images showing the interaction of ZH-1c-ENVs and ENVs with microglial cells (scale bars: 25 μm). (E) Quantitative fluorescence analysis of the binding efficiency of ZH-1c-ENVs and ENVs with microglial cells. Data in B, C, E are presented as mean ±SD. ENVs: Extracellular nanovesicles.

***Additional Figure 3:*** *Evaluation of the in vitro and in vivo biocompatibility of ZH-1c-ENVs@Dex.*

Additional Figure 3 Evaluation of the *in vitro* and *in vivo* biocompatibility of ZH-1c-ENVs@Dex.
(A) Schematic diagram of the experimental procedures for *in vitro* and *in vivo* biocompatibility assessment. (B) Cell viability of BV2 cells after co-incubation with ZH-1c-ENVs@Dex at different Dex concentrations, determined using the CCK-8 assay. (C) Live/dead fluorescence staining for cell viability verification (scale bars: 25 μm). (D) Changes in mouse body weight after injection of ZH-1c-ENVs@Dex. (E, F) Blood biochemical analysis of liver (alanine aminotransferase [ALT] and aspartate aminotransferase [AST]) and kidney (blood urea nitrogen [BUN], creatinine [Cr]) function indicators in mice. (G) Hematoxylin and eosin staining of major mouse organs (liver, kidney, spleen, heart, lung) to observe pathological changes in tissue structure (scale bars: 100 μm). Data in B, D-F are presented as mean ±SD. Experiments were performed in triplicate; *n* = 6 animals per group. CCK-8: Cell Counting Kit-8; Cr: creatinine; Dex: dexmedetomidine; ENVs: extracellular nanovesicles.

***Additional Figure 4:*** *In vitro preparation and validation of A*β *oligomers and fibrils.*

Additional Figure 4 *In vitro* preparation and validation of Aβ oligomers and fibrils.
(A) Western blot and transmission electron microscopy validation of *in vitro*-prepared Aβ oligomers and fibrils. (B) Immunofluorescence detection of the microglial activation marker Iba1. Scale bars: 25 μm. Data in B are presented as mean ± SD. All experiments were performed in triplicate. ^***^P < 0.001. Aβ: Amyloid-beta; DAPI: 4ʹ,6-diamidino-2-phenylindole; Iba1: ionized calcium-binding adapter molecule 1.

***Additional Figure 5:*** *Effects of ZH-1c-ENVs@Dex on lysosomal activity in microglial cells.*

Additional Figure 5 Effects of ZH-1c-ENVs@Dex on lysosomal activity in microglial cells.
(A) Western blot analysis of Lamp1, CatB, and V-ATPase expression in microglial cells across different groups. (B) LysoSensor staining to detect the total number of lysosomes in microglial cells across different groups; scale bars: 25 μm. Data are presented as mean ± SD. Experiments were repeated three times. ^*^P < 0.05, ^**^P < 0.01, ^***^P < 0.01. Aβ: Amyloid-beta; CatB: cathepsin B; DAPI: 4ʹ,6-diamidino-2-phenylindole; Dex: dexmedetomidine; ENVs: extracellular nanovesicles.

***Additional Figure 6:*** *Effects of ZH-1c-ENVs@Dex on Sirt3 expression and activity in AD mice.*

Additional Figure 6 Effects of ZH-1c-ENVs@Dex on Sirt3 expression and activity in AD mice.
(A) Time-lapse imaging of fluorescein isothiocyanate-labeled Aβ1-42 uptake and clearance in BV2 cells. (B, C) WB analysis of Sirt3 expression levels in hippocampal tissues across different groups. (D) Fluorescence assay to measure Sirt3 activity. (E) Transmission electron microscopy images of mitochondrial morphology in each group (scale bar = 500/200 nm). Data in C and D are presented as mean ±SD. ^**^P < 0.01; *n* = 3 independent experiments. AD: Alzheimer’s disease; Dex: dexmedetomidine; ENVs: extracellular nanovesicles.

***Additional Figure 7:*** *Effects of ZH-1c-ENVs@Dex on cognitive function in AD mice.*

Additional Figure 7 Effects of ZH-1c-ENVs@Dex on cognitive function in AD mice.
(A) Escape latency in the MWM test across different groups. (B) Number of platform crossings at 6 weeks post-treatment; (C) Percentage of time spent in the target quadrant at 6 weeks. (D) Representative swimming paths of mice in each group at 6 weeks. Data in A-C are presented as mean ± SD. ^***^P < 0.001; *n* = 5 animals per group. AD: Alzheimer’s disease; Dex: dexmedetomidine; ENVs: extracellular nanovesicles.

***Additional Table 1:*** *Complete blood count.*

## Data Availability

*The datasets generated and/or analyzed during the current study are not publicly available due to privacy and confidentiality agreements with the participants but are available from the corresponding author on reasonable request.*
